# Cytotoxic Serrulatane-Type Diterpenoids from the Gorgonian *Euplexaura* sp. and Their Absolute Configurations by Vibrational Circular Dichroism

**DOI:** 10.1038/s41598-017-12841-2

**Published:** 2017-10-02

**Authors:** Fei Cao, Chang-Lun Shao, Yun-Feng Liu, Hua-Jie Zhu, Chang-Yun Wang

**Affiliations:** 1 School of Medicine and Pharmacy, Ocean University of China; Key Laboratory of Marine Drugs, The Ministry of Education of China, Qingdao, 266003 China; 2 Key Laboratory of Pharmaceutical Quality Control of Hebei Province, College of Pharmaceutical Sciences, Hebei University, Baoding, 071002 China; 3 Laboratory for Marine Drugs and Bioproducts, Qingdao National Laboratory for Marine Science and Technology, Qingdao, 266071 China; 4 Institute of Evolution & Marine Biodiversity, Ocean University of China, Qingdao, 266003 China

## Abstract

Vibrational circular dichroism (VCD) method has become robust and reliable alternative for the stereochemical characterization of natural products. In this paper, three new serrulatane-type diterpenoids, euplexaurenes A–C (**1**–**3**), and a known metabolite, anthogorgiene P (**4**), were obtained from the South China Sea gorgonian *Euplexaura* sp. GXWZ-05. The absolute configuration of C-11 in **1**–**4**, which was difficult to be determined by common means due to the high conformational flexibility of the eight-carbon aliphatic chain attached at C-4, was determined by VCD method, suggesting a new horizon to define the absolute configurations of natural products possessing chains. Compounds **1**–**4** were found to show selective cytotoxic activities against human laryngeal carcinoma (Hep-2) cell line with the IC_50_ values of 1.95, 7.80, 13.6 and 5.85 *μ*M, respectively.

## Introduction

In the pharmaceutical chemistry and related fields, the absolute configuration is of prime importance in the interaction of drugs and organisms, since all receptors in the human body are chiral and probably exhibit different pharmacologic effects and pharmacokinetics between two enantiomers^1^. However, the determination of the absolute configurations for chiral natural products is one of the most challenge for natural product chemists. In current natural products research, X-ray diffraction and chiroptical methods are the most important and popular tools for determining the absolute configurations of novel natural products^1,2^. While, natural products are commonly available in small amounts from natural sources and usually do not bear heavy atoms, which often prevent direct assignment of the absolute configurations by X-ray diffraction method^2^. Vibrational circular dichroism (VCD) is one of the chiroptical method which, if combined with accurate quantum mechanical calculations, offers a powerful approach to the determination of absolute configurations in chiral natural products^3–6^. Interesting fact is VCD method has become robust and reliable alternative for the stereochemical characterization of natural products, especially in conditions not accessible to other methods.

**Figure 1:**
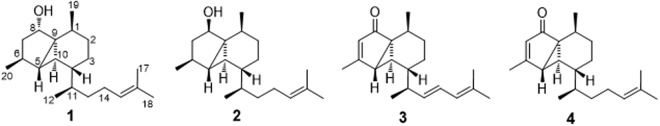
Chemical structures of 1–4.

Recently, in our continuing efforts to discover new bioactive substances with complicated absolute configurations from the South China Sea corals^7–11^, the gorgonian *Euplexaura* sp. GXWZ-05 attracted our attention due to the cytotoxic activity of its EtOAc extract. As a result, four serrulatane-type diterpenoids (Fig. 1), including three new compounds, euplexaurenes A–C (**1**–**3**), and a known compound, anthogorgiene P (**4**)^12^, were isolated. In order to determine the absolute configurations of **1**–**4**, VCD chiroptical method was applied. Herein, we report the isolation and absolute configurations of the new compounds, as well as the cytotoxic activities of **1**–**4**.

## Results and Discussion

**Figure 2:**
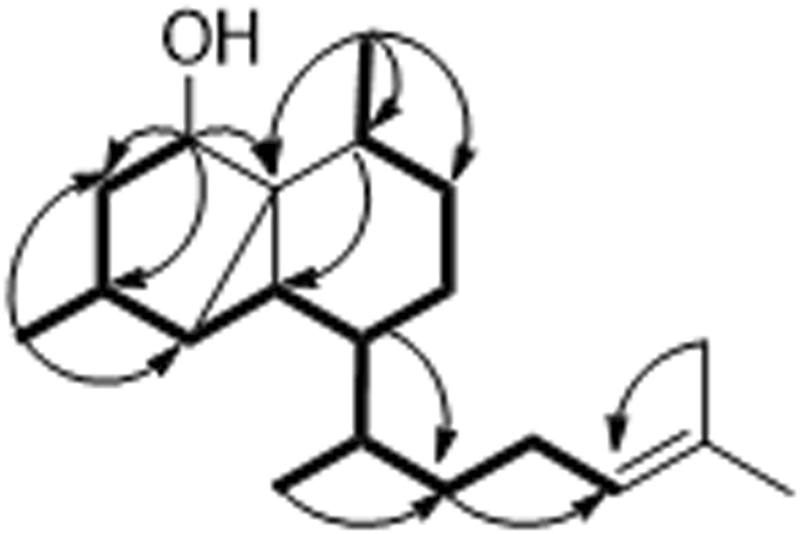
COSY and key HMBC correlations of 1.

Euplexaurene A (**1**) was a colorless oil with the molecular formula of C_20_H_34_O (four degrees of unsaturationon) on the basis of positive HRESIMS. The trisubstituted double bond [*δ*_H_ 5.12 (1 H, t, *J* = 6.5 Hz); *δ*_C_ 131.1 and 125.0] could account for one of the four degrees of unsaturation in **1**. Thus, a tricyclic nucleus was required for **1**. In the ^1^H NMR spectrum, five methyl signals with a singlet or doublet at *δ*_H_ 1.69 (3 H, s), 1.62 (3 H, s), 0.98 (3 H, d, *J* = 6.5 Hz), 0.96 (3 H, d, *J* = 7.5 Hz), and 0.92 (3 H, d, *J* = 7.0 Hz) were observed (Table 1). In the ^13^C NMR spectrum, 20 carbon signals assignable to two quaternary carbons, eight methines, five methylenes, and five methyls were revealed (Table 1). The above spectroscopic data suggested that **1** should be a serrulatane-type diterpenoid^12–15^. However, the traditional serrulatane-type diterpenoids nuclei only included a bicyclic system, which was not in accordance with the derived structure of the tricyclic nucleus in **1**. Thus, a new connectivity type must be existed in **1** to form a novel tricyclic system. In the HMBC spectrum of **1**, the correlations from H-5 to C-1 and C-8 (Fig. 2) suggested the direct connection between C-5 and C-9 forming a 5,3,6-tricyclic unit of serrulatane-type diterpenoids. In fact, only one compound with this moiety (**4**)^12^ was obtained from nature. It has been postulated that the 5,3,6-tricyclic moiety of the serrulatane-type diterpenoids was derived from bicyclic system of the traditional serrulatane-type diterpenoid via aromatic rearrangement^12^. In fact, the NMR spectra of **1** and **4** were similar. The main difference between **1** and **4** in the ^1^H NMR spectra was the presence of an oxygen-bearing methine doublet at *δ*_H_ 4.36 [1 H, t (8.0), H-8] in **1** instead of an olefinic methine singlet at *δ*_H_ 5.39 (1 H, s, H-7) in **4**. Accordingly, in the ^13^C NMR spectrum of **1** the signal of one oxygen-bearing methine (*δ*_C_ 75.0), one methine (*δ*_C_ 33.9) and one methylene (*δ*_C_ 38.4) were observed in place of the signals of carbonyl carbon (*δ*_C_ 209.0) and trisubstituted double bond (*δ*_C_ 178.0 and 123.5) in **4**, respectively. The above NMR data suggested that the *α*, *β*-unsaturated ketone group in **4** was hydrogenated in **1**, confirmed by the HMBC cross-peaks from H-8 to C-6, C-7, C-9, and from H-20 to C-5 and C-7 (Fig. 2). Detailed analysis of the 2D NMR spectra of **1** allowed the assignment for all of the proton and carbon resonances.

**Table 1:** NMR spectroscopic data for compound 1^a^. ^a^Spectra measured at 500 MHz in CDCl_3_.

No.	^1^H	^13^C	COSY	Key HMBC
1	2.08, m	32.7, CH	H-2, H-19	C-3, C-10
2	1.63, m	31.6, CH_2_	H-1, H-3	C-4, C-9
	0.53, m			
3	1.34, m	26.2, CH_2_	H-2, H-4	C-1, C-10
	0.79, m			
4	1.06, m	42.4, CH	H-3, H-10, H-11	C-2, C-13
5	1.92, m	27.7, CH	H-6, H-10	C-4, C-20
6	0.88, m	33.9, CH	H-5, H-7, H-20	C-8, C-20
7	1.96, m	38.4, CH_2_	H-6, H-8	C-5, C-9
	0.73, m			
8	4.36, t (8.0)	75.0, CH	H-7	C-6, C-7, C-9
9	—	39.5, C	—	—
10	0.72, m	38.6, CH	H-4, H-5	C-1, C-3
11	1.47, m	38.4, CH	H-4, H-12, H-13	C-3, C-10, C-14
12	0.92, d (7.0)	16.7, CH_3_	H-11	C-4, C-13
13	1.53, m	34.3, CH_2_	H-11, H-14	C-4, C-12, C-15
	1.22, m			
14	2.05, m	25.9, CH_2_	H-13, H-15	C-11, C-16
	1.92, m			
15	5.12, t (6.5)	125.0, CH	H-14	C-13, C-17, C-18
16	—	131.1, C	—	—
17	1.62, s	17.7, CH_3_	—	C-15
18	1.69, s	25.7, CH_3_	—	C-15
19	0.98, d (6.5)	18.1, CH_3_	H-1	C-1, C-2, C-9
20	0.96, d (7.5)	18.8, CH_3_	H-6	C-5, C-6, C-7

**Figure 3:**
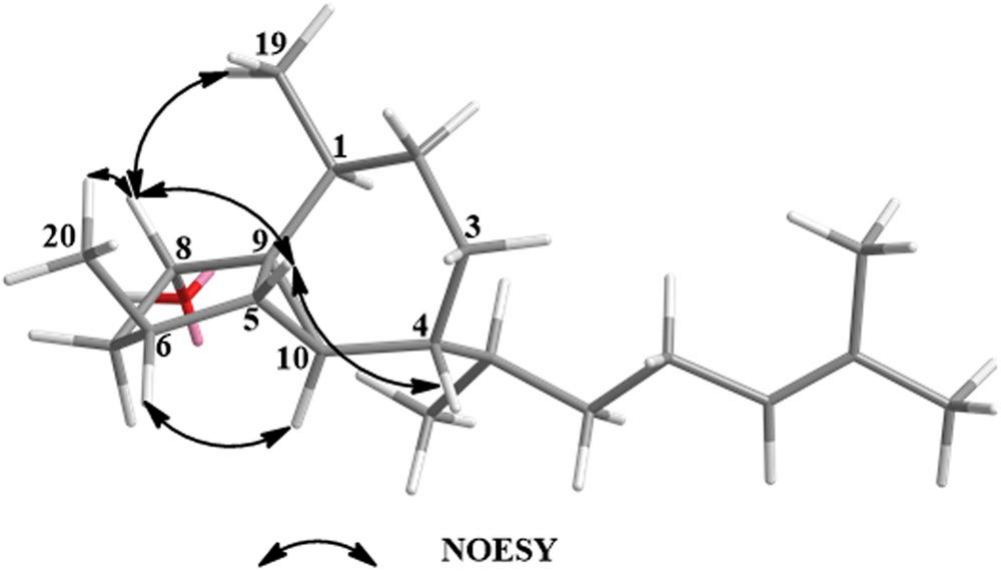
Selected NOESY correlations of 1.

**Figure 4:**
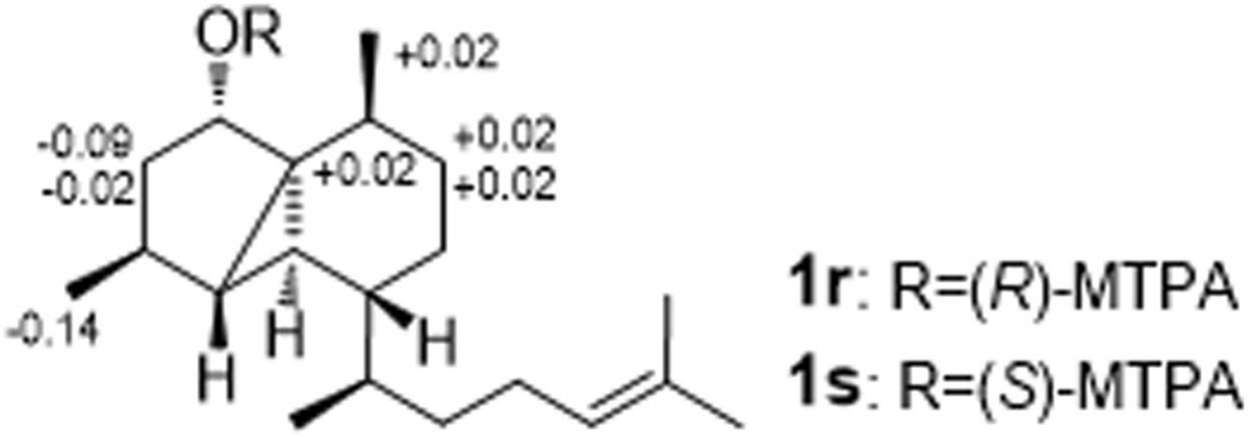
Values of Δ*δ*_H(*S*-*R*)_ (measured in CD_3_OD) of the MTPA esters of 1.

The relative configuration of the 5,3,6-tricyclic unit in **1** was deduced by NOESY experiments (Fig. 3). In the NOESY spectrum of **1**, H-8 was found to show NOESY correlations with H-5, H_3_-19 and H_3_-20, and the NOESY correlation could be observed between H-4 and H-5, indicated that these protons should be on the same face of **1**. On the opposite face of **1**, the key NOESY cross-peaks between H-6 and H-10 suggested that these two protones should be cofacial. To determine the absolute configuration of **1**, we applied modified Mosher’s method using (*R*)-( + )- and (*S*)-(−)-MTPA-Cl to give the (*S*)- and (*R*)-MTPA esters of **1** (**1 s** and **1r**), respectively. The absolute configuration at C-8 in **1** was assigned as *S* deduced from the Δ*δ*_H_ values between the two MTPA esters (Fig. 4) following the MTPA rules^16^. Thus, the configuration of tricyclic nucleus for compound **1** was determined as 1 *S*, 4 *R*, 5 *R*, 8 *S*, 9 *R*, 10 *S*.

Euplexaurene B (**2**) was deduced to have the same molecular formula C_20_H_34_O as **1** by HRESIMS analysis. The^1^H NMR spectra of **2** (Table 2) and 1 were almost identical, which suggested that they may be a pair of epimers. When comparing their NMR spectra, the signals attributable to H-8 were found to be slightly different (*δ*_H_ 4.08 (1 H, d, *J* = 5.5 Hz), *δ*_C_ 81.5 in **2** *vs δ*_H_ 4.36 (1 H, t, *J* = 8.0 Hz), *δ*_C_ 75.0 in **1**), indicating that the noticeable difference between the epimers was the isomerization of C-8. This was supported by the NOESY crosspeaks of H-10/H-8. Hence, **2** is the 8-*epi*-isomer of **1**.

**Table 2:** NMR spectroscopic data for compound 2^a^. ^a^Spectra measured at 500 MHz in CDCl_3_.

No.	^1^H	^13^C	COSY	Key HMBC
1	2.49, m	33.0, CH	H-2, H-19	C-3, C-9
2	1.67, m	33.3, CH_2_	H-1, H-3	C-4, C-9
	0.52, m			
3	1.38, m	26.0, CH_2_	H-2, H-4	C-1, C-2, C-10
	0.76, m			
4	0.91, m	43.0, CH	H-3, H-10, H-11	C-2, C-13
5	2.01, m	28.3, CH	H-6, H-10	C-4, C-20
6	1.00, m	34.3, CH	H-5, H-7, H-20	C-5, C-8
7	1.51, m	40.8, CH_2_	H-6, H-8	C-5, C-20
	1.16, m			
8	4.08, d (5.5)	81.5, CH	H-7	C-6, C-7, C-9
9	0.79, m	40.8, C	—	—
10	—	40.8, CH	H-4, H-5	C-1, C-3
11	1.38, m	38.4, CH	H-4, H-12, H-13	C-3, C-12, C-14
12	0.89, d (6.5)	16.9, CH_3_	H-11	C-4, C-13
13	1.56, m	34.2, CH_2_	H-11, H-14	C-12, C-15
	1.17, m			
14	2.03, m	25.9, CH_2_	H-13, H-15	C-11, C-13, C-16
	1.90, m			
15	5.11, t (6.5)	125.0, CH	H-14	C-16, C-17, C-18
16	—	131.7, C	—	—
17	1.61, s	17.7, CH_3_	—	C-15
18	1.69, s	25.7, CH_3_	—	C-15
19	1.01, d (6.5)	18.0, CH_3_	H-1	C-1, C-2, C-9
20	1.11, d (7.0)	23.0, CH_3_	H-6	C-5, C-6, C-7

Euplexaurene C (**3**) was found to have a molecular formula of C_20_H_28_O with seven degrees of unsaturation based on HRESIMS, revealing the loss of two hydrogen protons compared with that of **4**. The ^1^H and ^13^C NMR data (Table 3) revealed that **3** should have the same structural features as those presenting in **4** except for the presence of a disubstituted double bond at C-13 and C-14 on the side chain in **3**. The position of the double bond was confirmed by the HMBC correlations from H-11 to C-14, and from H-15 to C-13. The coupling constant between H-13 and H-14 (16.0 Hz) of **3** defined the double bond to be in the *E* configuration. Thus, the planar structure of **3** was assigned as a 22,23-dehydro analogue of **4**. The ECD profile of **3** was similar to that of **4** (Fig. S2), suggesting the same (1 *S*, 4 *R*, 5 *R*, 9 *R*, 10 *S*) absolute configuration.

**Table 3:** NMR spectroscopic data for compound 3^a^. ^a^Spectra measured at 500 MHz in CDCl_3_.

No.	^1^H	^13^C	COSY	Key HMBC
1	2.47, m	26.4, CH	H-2, H-19	C-3, C-9
2	1.80, m	30.4, CH_2_	H-1, H-3	C-1, C-4, C-9
	0.71, m			
3	1.43, m	25.8, CH_2_	H-2, H-4	C-2, C-10
	0.94, m			
4	1.24, m	43.3, CH	H-3, H-10, H-11	C-9, C-11
5	1.95, m	35.4, CH	H-10	C-4, C-20
6	—	178.2, C	—	—
7	5.37, brs	123.7, CH	—	C-5, C-20
8	—	209.1, C	—	—
9	—	43.0, C	—	—
10	1.34, m	54.4, CH	H-4, H-5	C-3, C-6
11	2.45, m	38.0, CH	H-4, H-12, H-13	C-3, C-14
12	0.87, d (7.0)	16.6, CH_3_	H-11	C-4, C-13
13	5.54, d (16.0)	135.0, CH	H-11, H-14	C-12, C-15
14	5.62, dd (16.0, 6.0)	130.7, CH	H-13, H-15	C-11, C-16
15	5.01, d (6.0)	114.4, CH	H-14	C-16, C-17, C-18
16	—	123.6, C	—	—
17	1.34, s	24.5, CH_3_	—	C-15
18	1.32, s	24.2, CH_3_	—	C-15
19	0.93, d (6.0)	19.8, CH_3_	H-1	C-1, C-9
20	2.15, s	18.9, CH_3_	H-6	C-5, C-6, C-7

**Figure 5:**
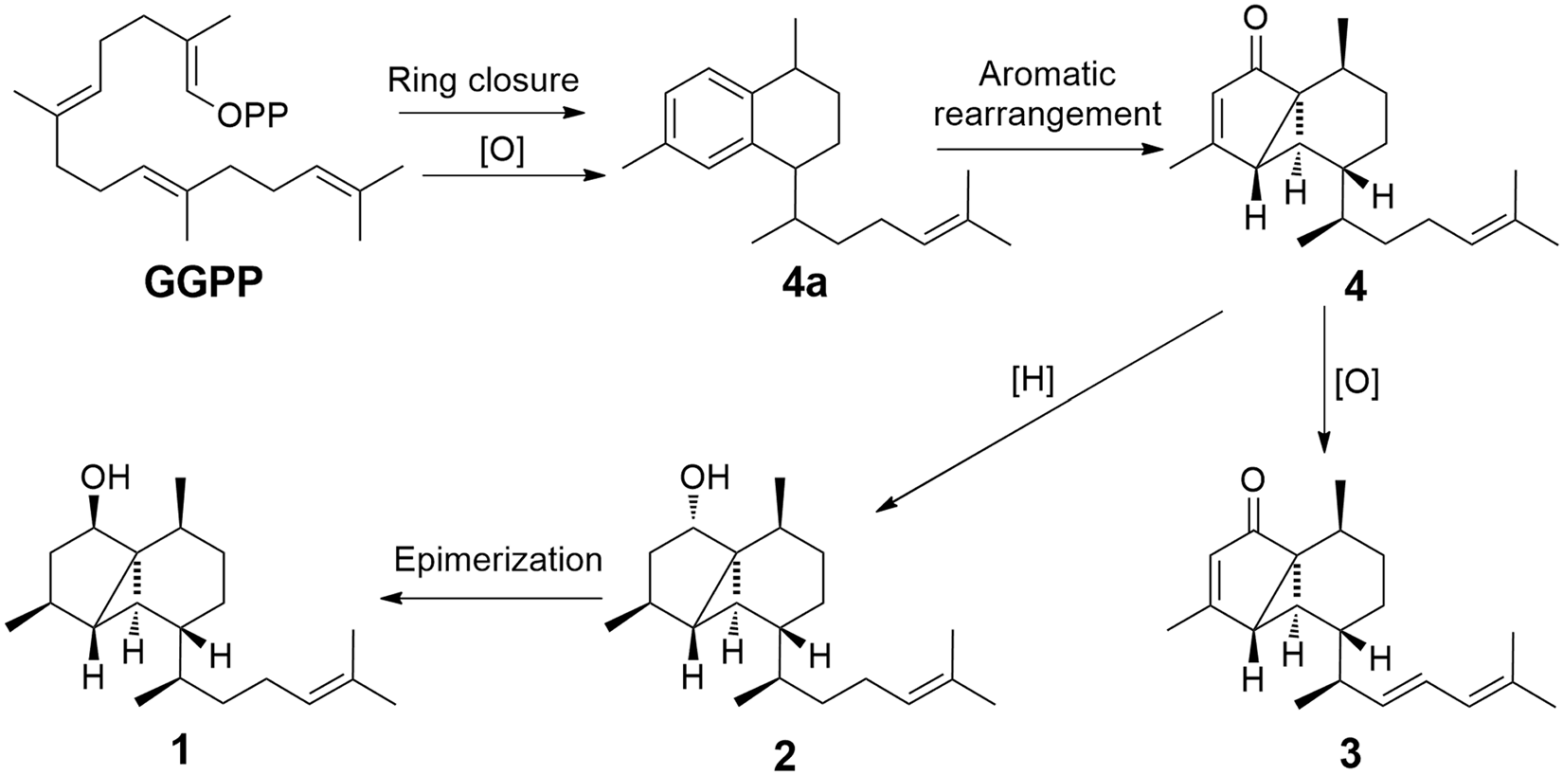
Proposed Biogenetic Pathways for 1–4.

Although serrulatane-type diterpenoids have been isolated from marine organisms^12–15^, they were mainly appeared in the form of bicyclic system^13–15^. Anthogorgiene P (**4**) was firstly isolated as a novel skeleton compound from a Chinese gorgonian *Anthogorgia* sp.^12^. Euplexaurenes A–C (**1**–**3**) represent the serrulatane-type diterpenoids characterized with a 5,3,6-tricyclic skeleton isolated from nature for the second time. A hypothesized biosynthetic pathway of **1**–**4** starting from geranylgeranyl pyrophosphat (GGPP) was proposed (Fig. 5). A key intermediate (**4a**) in the biosynthesis derives from GGPP by a ring closure and oxidation. Anthogorgiene P (**4**) is formed from **4a** via aromatic rearrangement to form an unusual 5,3,6-tricyclic nucleus. Then, **4** is oxidized to form euplexaurene C (**3**), and hydrated to form euplexaurene B (**2**), respectively. Finally, the epimerization of **2** gives euplexaurene A (**1**). The new structural patterns found from this gorgonian specimen implied the presence of new biogenetic pathways within marine organisms to adopt different ecological environments.

The absolute configuration of C-11 in serrulatane-type diterpenoid was difficult to be determined by common means of NMR and ECD methods due to the high conformational flexibility of the eight-carbon aliphatic chain attached at C-4. In previous study, the stereochemistry of **4** was not assigned completely^12^. Meanwhile, the eight-carbon aliphatic chain as in **1**–**4** has been frequently found in terpenoids, ranging from bisabolene sesquiterpenes such as perezone^17^, to sterols such as desmosterol^18^, which was the last biogenetic intermediate in the biosynthesis of cholesterol^19^.

**Figure 6:**
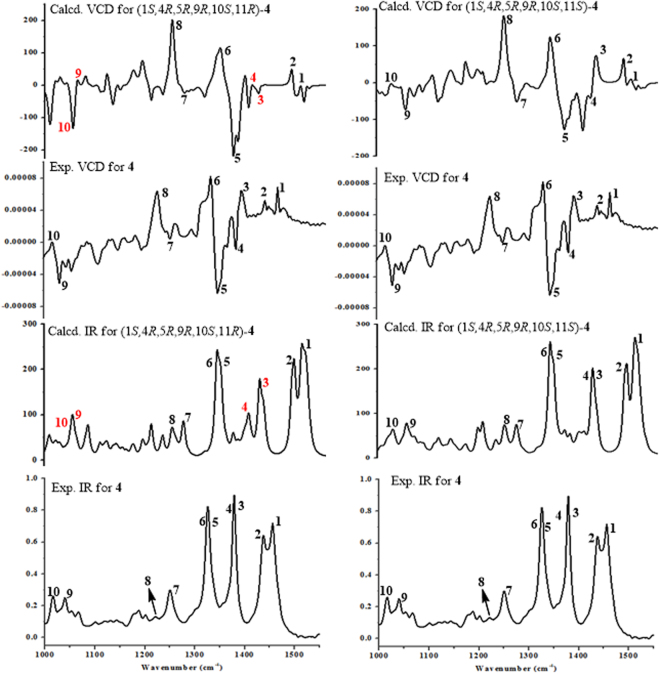
The observed and simulated VCD and IR for 4.

VCD spectroscopy is one such chiroptical technique that sheds new light on many important phenomena studies intensively. The interplay of VCD spectra of chiral molecules in the liquid state and computational studies has led to a remarkably detailed picture of the systems. In recent years, this technique has provided a powerful physicochemical method for the assignment of absolute configurations in natural products. Especially, compared to the other chiroptical methods, VCD present many advantages, since it could be applied to virtually any molecules without the requirement of either UV or Vis chromophores. In present research, VCD has opened a new horizon to define the absolute configurations at C-11 in **1**–**4**. As the low yields of **1**–**3**, compound **4** was chosen to test its experimental VCD spectrum. Thus, the two C-11 diastereomers of **4** were investigated by quantum chemical TDDFT calculations of their VCD spectra. Conformational searches were performed using MMFF94S force field for (1 *S*,4 *R*,5 *R*,9 *R*,10 *S*,11 *S*)-**4** and (1 *S*,4 *R*,5 *R*,9 *R*,10 *S*,11 *R*)-**4**. All geometries (78 lowest energy conformers for (1 *S*,4 *R*,5 *R*,9 *R*,10 *S*,11 *S*)-**4** and 30 for (1 *S*,4 *R*,5 *R*,9 *R*,10 *S*,11 *R*)-**4**, respectively) with relative energy from 0‒10 kcal/mol were used in optimizations at the B3LYP/6-31 G(d) level using Gaussian09 package^20^. The B3LYP/6-31 G(d)-optimized conformers (19 lowest energy conformers for (1 *S*,4 *R*,5 *R*,9 *R*,10 *S*,11 *S*)-**4** and 10 for (1 *S*,4 *R*,5 *R*,9 *R*,10 *S*,11 *R*)-**4**, respectively; see Supporting Information for details) with relative energy from 0 to 4.6 kcal/mol were then re-optimized at the B3LYP/6-311 + G(d) level. The IR and VCD frequencies were calculated for all these structures at the B3LYP/6-311 + G(d) level, and the conformational populations were obtained by means of the Δ*G* = −*RT* ln*K* equation to generate the Boltzmann-averaged IR and VCD spectra. The experimental IR and VCD spectra were measured in CDCl_3_ at room temperature. The comparison of the two VCD spectra was superimposed in Fig. 6.

All of the calculated IR signals of (1 *S*,4 *R*,5 *R*,9 *R*,10 *S*,11 *S*)-**4** had agreements with the experimental IR signals, while the signals of 3, 4, 9, and 10 in the calculated IR spectrum of (1 *S*,4 *R*,5 *R*,9 *R*,10 *S*,11 *R*)-**4** had disagreements (the signals labeled in red) (Fig. 6) with the corresponding signals in the experimental spectrum. This suggested that the structure of (1 *S*,4 *R*,5 *R*,9 *R*,10 *S*,11 *S*)-**4** was closer to the real case. Furthermore, the calculated VCD spectra were compared with the experimental VCD spectrum, respectively. Most of the calculated VCD signals of (1 *S*,4 *R*,5 *R*,9 *R*,10 *S*,11 *R*)-**4** had the identity with the experimental results, however, the signals of 3, 4, 9, and 10 (the signals labeled in red) (Fig. 6) did not match the experimental signals. For (1 *S*,4 *R*,5 *R*,9 *R*,10 *S*,11 *S*)-**4**, the calculated VCD spectrum compared well with the experimental VCD spectrum. Therefore, based on the IR and VCD calculations, the absolute configuration at C-11 of **4** was determined to be *S*. Obviously, calculations of the VCD spectra for conformational studies may be a promising and growing field for structural investigation of natural chiral molecules.

The cytotoxic activities of **1**–**4** were evaluated against a panel of human tumor cell lines (Hep-2, HL-60, K562, HeLa, and HCT-116), and a non tumoral cell line, rat kidney cell (NRK-52E). All of the tested compounds (**1**–**4**) exhibited selective cytotoxic activities against Hep-2 cells with the IC_50_ values of 1.95, 7.80, 13.6 and 5.85 *μ*M, respectively. Interestingly, **1**–**4** exhibited no cytotoxicity to the other cell lines (IC_50_ > 10.0 *μ*M). Especially, **1**–**4** displayed no cytotoxicity to NRK-52E cell line (IC_50_ > 100 *μ*M). Preliminary structure–activity analysis suggested that the hydroxy group at C-8 may increase the cytotoxic activity, and the presence of 8*α*-OH contributed more to the activity than 8*β*-OH. In addition, the antibacterial activities of **1**–**4** were also tested toward several pathogenic bacteria. But none of the tested compounds showed any activity (MIC > 25.0 *μ*M).

In conclusion, four serrulatane-type diterpenoids (**1**–**4**) with potent cytotoxicity against Hep-2 were isolated from the gorgonian *Euplexaura* sp. VCD experiment combined with accurate quantum mechanical calculation method was carried out to assign their absolute configurations. It could be concluded that VCD is one of important chiroptical methods for the structural elucidation of natural products.

## Methods

### General Experimental Procedures

Optical rotations were measured on an Optical Activity Limited AA-55 polarimeter. ECD spectra were obtained on a Bio-logic MOS-450 circular dichroism spectrometer. IR and VCD spectra were acquired using a BioTools Chiral*IR*-2X spectrophotometer. NMR spectra (500 MHz for^1^H NMR and 125 MHz for ^13^C NMR) were measured on a Bruker AV-500 spectrometer. ESIMS spectra were obtained using a Micromass Q-TOF spectrometer. Preparative HPLC was performed on a Shimadzu LC-20AT HPLC system with a SPD-M20A detector using a Waters C_18_ semi-preparative column (250 × 19 mm, 5 *μ*m). Silica gel (200–300 mesh, Qing Dao Marine Chemical Inc.), Sephadex LH-20 (Pharmacia, Co.) and ODS (40–63 mm, Octadecyl silica, YMC, Kyoto, Japan) were used for column chromatography. TLC was performed on plates with precoated silica gel GF_254_ (Yantai Zifu Chemical Group Co.).

### Animal Materials

The gorgonian samples of *Euplexaura* sp. was collected in the South China Sea at a depth of 18–25 m in April 2011 from Weizhou Island sea area, China, which was identified by Dr. Xiubao Li, South China Sea Institute of Oceanology, Chinese Academy of Sciences. A voucher specimen (GXWZ-05) has been deposited at the Key Laboratory of Marine Drugs, Ministry of Education, Ocean University of China, Qingdao, China.

### Extraction and Isolation

Specimens of *Euplexaura* sp. (GXWZ-05) (1780 g, wet weight) was chopped and exhaustively macerated with 95% EtOH (6 × 2.0 L). The EtOH solution was concentrated under reduced pressure to provide a crude extract (9.5 g), which was further partitioned between H_2_O and EtOAc to offer EtOAc extract (3.0 g). This extract was subjected to silica gel column chromatography using a mixture of petroleum ether (PE)/EtOAc (9:1 → 1:9). The main fractions were subjected to Sephadex LH-20 chromatography eluting with mixtures of PE/CH_2_Cl_2_/MeOH = 2:1:1 and CH_2_Cl_2_/MeOH = 1:1, and then futher isolated by preparative HPLC using a C_18_ column at a flow rate of 4.0 mL/min (MeCN/H_2_O, 80:20; UV detection at 210 nm) to provide **1** (6.0 mg), **2** (4.5 mg), **3** (2.5 mg), and **4** (12.0 mg).

*Euplexaurene A* (***1***)*:* Colorless oil; [*α*]_D_^25^ =  + 44.9 (*c* 0.10, CH_3_OH); IR: 3432, 2928, 1726, 1456, 1374, 1029; ^1^H and ^13^C NMR data, see Table 1; positive HRESIMS *m/z* 291.2677 ([M + H]^+^, C_20_H_35_O; calc. 291.2682).

*Euplexaurene B* (***2***)*:* Colorless oil; [*α*]_D_^25^ =  + 63.1 (*c* 0.10, CH_3_OH); IR: 3437, 2953, 1720, 1462, 1361, 1042;^1^H and ^13^C NMR data, see Table 2; positive HRESIMS *m/z* 291.2676 ([M + H]^+^, C_20_H_35_O; calc. 291.2682).

*Euplexaurene C* (***3***)*:* Colorless oil; [*α*]_D_^23^ =  + 23.7 (*c* 0.05, CH_3_OH); IR: 2925, 1677, 1458, 1365, 1241, 1005; CD (MeOH) *λ* (mdeg): 228 (−55), 274 (90), 326 (−42) nm; ^1^H and ^13^C NMR data, see Table 3; positive HRESIMS *m/z* 285.2208 ([M + H]^+^, C_20_H_29_O; calc. 285.2213).

*Anthogorgiene P* (***4***)*:* Colorless oil; [*α*]_D_^23^ =  + 33.5 (*c* 0.05, CH_3_OH); IR: 2912, 1660, 1462, 1383, 1257, 1014; CD (MeOH) *λ* (mdeg): 229 (−79), 275 (107), 325 (−50) nm; positive HRESIMS *m/z* 287.2367 ([M + H]^+^, C_20_H_31_O; calc. 287.2369).

### Preparation of the MTPA Ester Derivatives of 1

Euplexaurene A (**1**) (2.0 mg) was divided into two same portions. Each sample (1.0 mg) was treated with (*R*)-MTPA-Cl (10 *μ*L) and (*S*)-MTPA-Cl (10 *μ*L) in pyridine (500 *μ*L) at room temperature, respectively. After 5 h, the solvents were removed under reduced pressure, and the residues were separated on a silica gel column chromatography with PE/EtOAc (5:1) to give the (*S*)-MTPA ester **1 s** and (*R*)-MTPA ester **1r**, respectively.

(*S*)*-MTPA ester* (***1 s***)*:* ^1^H NMR (CD_3_OD, 500 MHz) *δ*_H_ 7.55–7.40 (5 H, m, Ph), 5.66 (1 H, m, H-15), 4.41 (1 H, m, H-8), 3.54 (3 H, s, OCH_3_-MTPA), 2.27 (1 H, m, H-1), 2.11 (1 H, m, H-6), 1.61 (1 H, m, H-2a), 1.49 (1 H, m, H-7a), 1.16 (1 H, m, H-7b), 1.02 (3 H, d, *J* = 7.0 Hz, H_3_-19), 0.81 (3 H, d, *J* = 7.0 Hz, H_3_-20), 0.60 (1 H, m, H-2b); positive ESIMS *m/z* 529.4 [M + Na]^+^, 545.4 [M + K]^+^.

(*R*)*-MTPA ester* (***1r***)*:* ^1^H NMR (CD_3_OD, 500 MHz) *δ*_H_ 7.55–7.40 (5 H, m, Ph), 5.65 (1 H, m, H-15), 4.41 (1 H, m, H-8), 3.52 (3 H, s, OCH_3_-MTPA), 2.25 (1 H, m, H-1), 2.16 (1 H, m, H-6), 1.59 (1 H, m, H-2a), 1.58 (1 H, m, H-7a), 1.18 (1 H, m, H-7b), 1.00 (3 H, d, *J* = 7.0 Hz, H_3_-19), 0.95 (3 H, d, *J* = 7.0 Hz, H_3_-20), 0.58 (1 H, m, H-2b); positive ESIMS *m/z* 529.4 [M + Na]^+^, 545.4 [M + K]^+^.

### Computational Section

Quantum theory was well developed and used in energies calculations, analytic gradients, and true analytic frequencies study. For VCD calculation, time-dependent density functional theory (TD-DFT) was used. Before VCD calculation, all the conformers were optimized to ensure that all the conformers were the optimum structure with low energetics. Conformational searches were performed using MMFF94S force field. B3LYP/6-311 + G(d)//B3LYP/6-311 + G(d) method was used for VCD computations. After the calculations of VCD for each conformation, Boltzmann statistics was used to simulate their corresponding values, respectively. These simulated data were used to compare to experimental data.

### Cytotoxic Activity Assays

The cytotoxic activities of **1**–**4** against a panel of human tumor cell lines, Hep-2 (human laryngeal carcinoma), HL-60 (human promyelocytic leukemia), K562 (human erythroleukemia), HeLa (cervical cancer), and HCT-116 (human colon carcinoma), together with a non tumoral cell line, NRK-52E (normal rat kidney) were determined by using MTT method, according to the protocols described in the literature^21^.

### Antibacterial Assays

Antibacterial activity was evaluated by the conventional broth dilution assay^8^. Gram-positive bacteria (*Micrococcus lysodeikticus*, *Bacillus cereus*, *Bacillus megaterium*) and Gram-negative bacteria (*Proteusbacillm vulgaris*, *Vibrio anguillarum*, *Vibrio parahemdyticus*) were used, and ciprofloxacin was used as a positive control.

## Electronic supplementary material

Supporting Information
