# Supplementary material for: Cytotoxic Serrulatane-Type Diterpenoids from the Gorgonian Euplexaura sp. and Their Absolute Configurations by Vibrational Circular Dichroism
Source: Sci Rep. 2017 Oct 2;7:12548. doi: 10.1038/s41598-017-12841-2 (PMC5624892; doi:10.1038/s41598-017-12841-2)
Supplement: Supplementary file 1 — Supporting Information [file 41598_2017_12841_MOESM1_ESM.doc]

**Cytotoxic Serrulatane-Type Diterpenoids from the Gorgonian *Euplexaura* sp. and Their Absolute Configurations by Vibrational Circular Dichroism**

Fei Cao,^1,2^ Chang-Lun Shao,^1,3^,* Yun-Feng Liu,^1,2^ Hua-Jie Zhu,^2^ and Chang-Yun Wang^1,3,4^,*

^1^School of Medicine and Pharmacy, Ocean University of China; Key Laboratory of Marine Drugs, The Ministry of Education of China, Qingdao 266003, China. ^2^Key Laboratory of Pharmaceutical Quality Control of Hebei Province, College of Pharmaceutical Sciences, Hebei University, Baoding 071002, China. ^3^Laboratory for Marine Drugs and Bioproducts, Qingdao National Laboratory for Marine Science and Technology, Qingdao 266071, China. ^4^Institute of Evolution & Marine Biodiversity, Ocean University of China, Qingdao 266003, China.

*Correspondence and requests for materials should be addressed to C.-Y. W. (changyun@ouc.edu.cn) or C.-L. S. (shaochanglun@ouc.edu.cn)

**List of Supporting Information**

**Figure S1.** Calculated ECD spectrum of **4** th experimental ECD spectrum of **4**.

**Figure S2.** Experimental ECD spectra of **3** and **4**.

**Figure S3.** ^1^H NMR (500 MHz, CDCl_3_) spectrum of compound **1**

**Figure S4.** ^13^C NMR (125 MHz, CDCl_3_) spectrum of compound **1**

**Figure S5.** HMQC (CDCl_3_) spectrum of compound **1**

**Figure S6.** ^1^H-^1^H COSY (CDCl_3_) spectrum of compound **1**

**Figure S7.** HMBC (CDCl_3_) spectrum of compound **1**

**Figure S8.** NOESY (CDCl_3_) spectrum of compound **1**

**Figure S9.** Partial NOESY (CDCl_3_) spectrum of compound **1**

**Figure S10.** HRESIMS spectrum of compound **1**

**Figure S11.** ^1^H NMR (500 MHz, CDCl_3_) spectrum of compound **2**

**Figure S12.** ^13^C NMR (125 MHz, CDCl_3_) spectrum of compound **2**

**Figure S13.** HMQC (CDCl_3_) spectrum of compound **2**

**Figure S14.** ^1^H-^1^H COSY (CDCl_3_) spectrum of compound **2**

**Figure S15.** HMBC (CDCl_3_) spectrum of compound **2**

**Figure S16.** NOESY (CDCl_3_) spectrum of compound **2**

**Figure S17.** HRESIMS spectrum of compound **2**

**Figure S18.** ^1^H NMR (500 MHz, CDCl_3_) spectrum of compound **3**

**Figure S19.** ^13^C NMR (125MHz, CDCl_3_) spectrum of compound **3**

**Figure S20.** HMQC (CDCl_3_) spectrum of compound **3**

**Figure S21.** ^1^H-^1^H COSY (CDCl_3_) spectrum of compound **3**

**Figure S22** HMBC (CDCl_3_) spectrum of compound **3**

**Figure S23.** NOESY (CDCl_3_) spectrum of compound **3**

**Figure S24.** HRESIMS spectrum of compound **3**

**Figure S25.** ^1^H NMR (500 MHz, CDCl_3_) spectrum of compound **4**

**Figure S26.** ^13^C NMR (125 MHz, CDCl_3_) spectrum of compound **4**

**Figure S27.** HRESIMS spectrum of compound **4**

**Figure S28.** ^1^H NMR (500 MHz, CD_3_OD) spectrum of compound **1s**

**Figure S29.** **^1^**H-^1^H COSY (CD_3_OD) spectrum of compound **1s**

**Figure S30.** ESI-MS spectrum of compound **1s**

**Figure S31.** ^1^H NMR (500 MHz, CD_3_OD) spectrum of compound **1r**

**Figure S32.** **^1^**H-^1^H COSY (CD_3_OD) spectrum of compound **1r**

**Figure S33.** ESI-MS spectrum of compound **1r**

**Figure S34.** Ten lowest energy conformers for (1*S*,4*R*,5*R*,9*R*,10*S*,11*R*)-**4**

**Figure S35.** Ninteen lowest energy conformers for (1*S*,4*R*,5*R*,9*R*,10*S*,11*S*)-**4**

**Table S1.** Energy analysis for (1*S*,4*R*,5*R*,9*R*,10*S*,11*R*)-**4**

**Table S2.** Energy analysis for (1*S*,4*R*,5*R*,9*R*,10*S*,11*S*)-**4**

**Table S3.** NMR spectroscopic data for compound **4**.


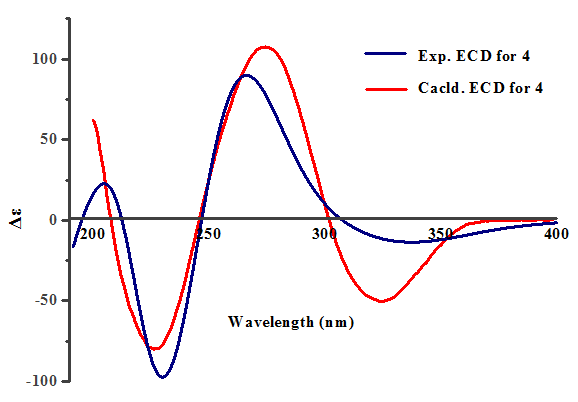


**Figure S1.** Calculated ECD spectrum of **4** th experimental ECD spectrum of **4**.


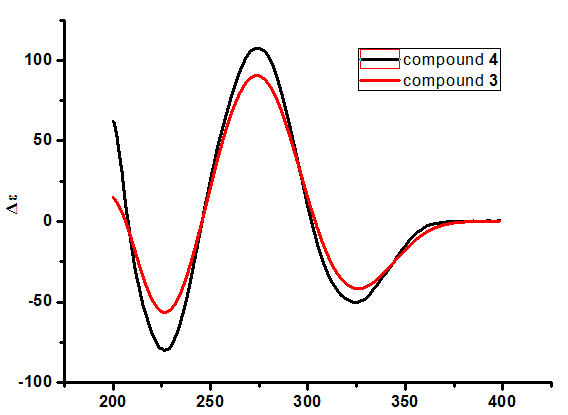


**Figure S2.** Experimental ECD spectra of **3** and **4**.


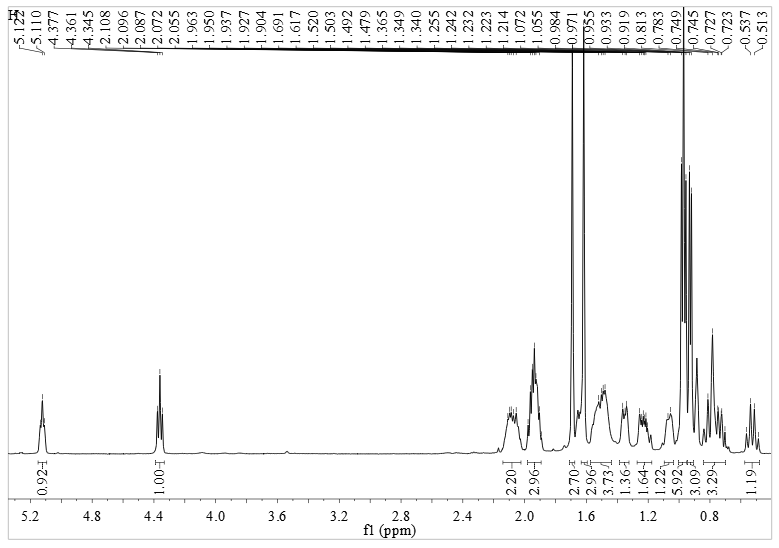


**Figure S3.** ^1^H NMR (500 MHz, CDCl_3_) spectrum of compound **1**


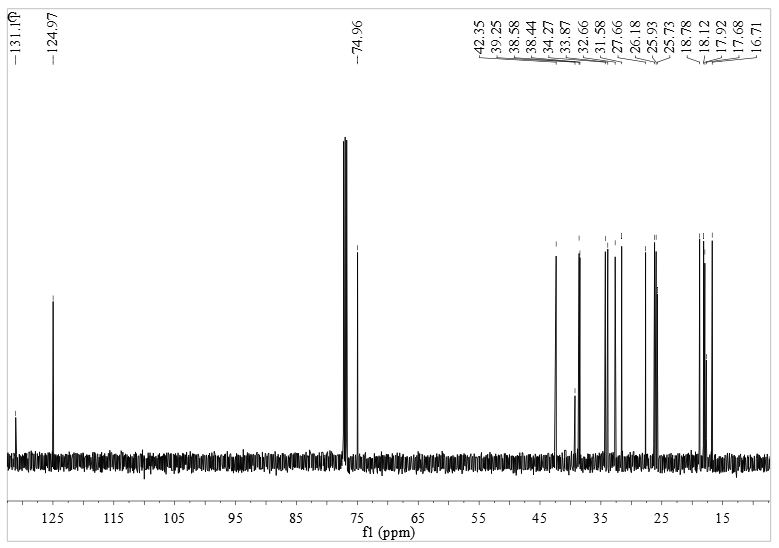


**Figure S4.** ^13^C NMR (125 MHz, CDCl_3_) spectrum of compound **1**


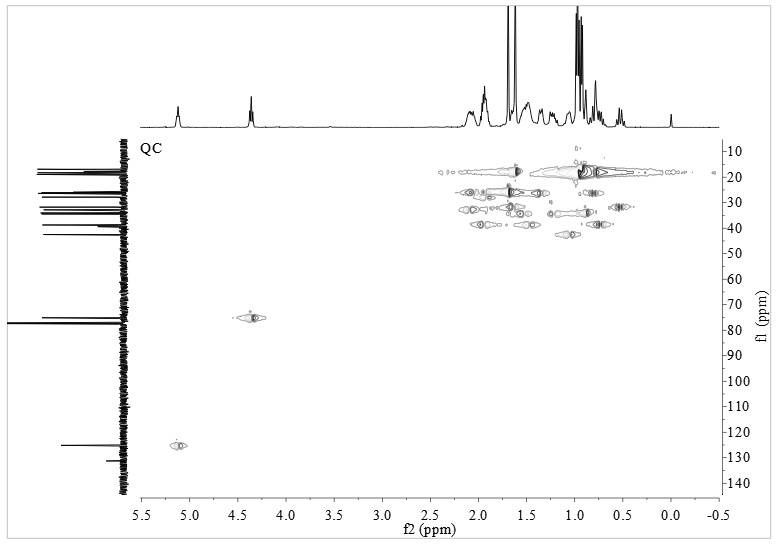


**Figure S5.** HMQC (CDCl_3_) spectrum of compound **1**


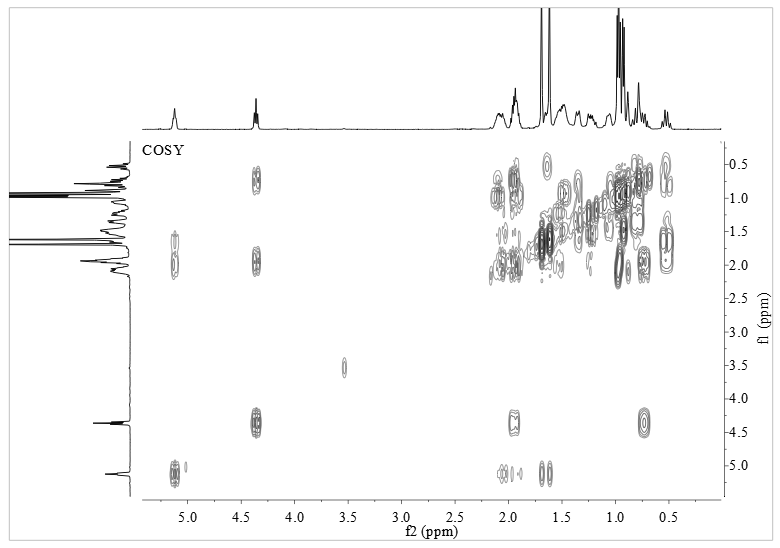


**Figure S6.** ^1^H-^1^H COSY (CDCl_3_) spectrum of compound **1**


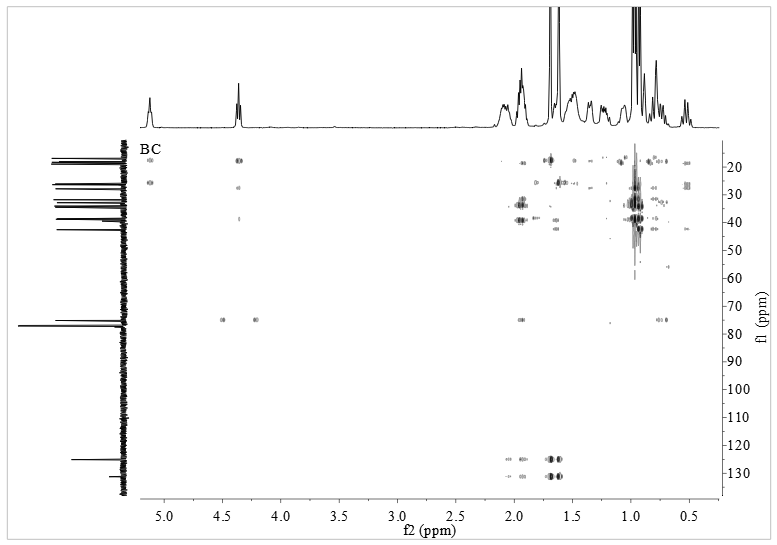


**Figure S7.** HMBC (CDCl_3_) spectrum of compound **1**


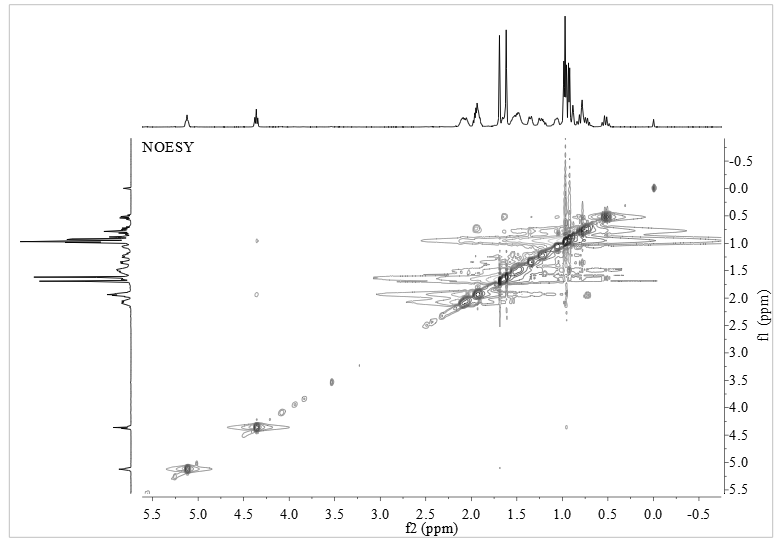


**Figure S8.** NOESY (CDCl_3_) spectrum of compound **1**


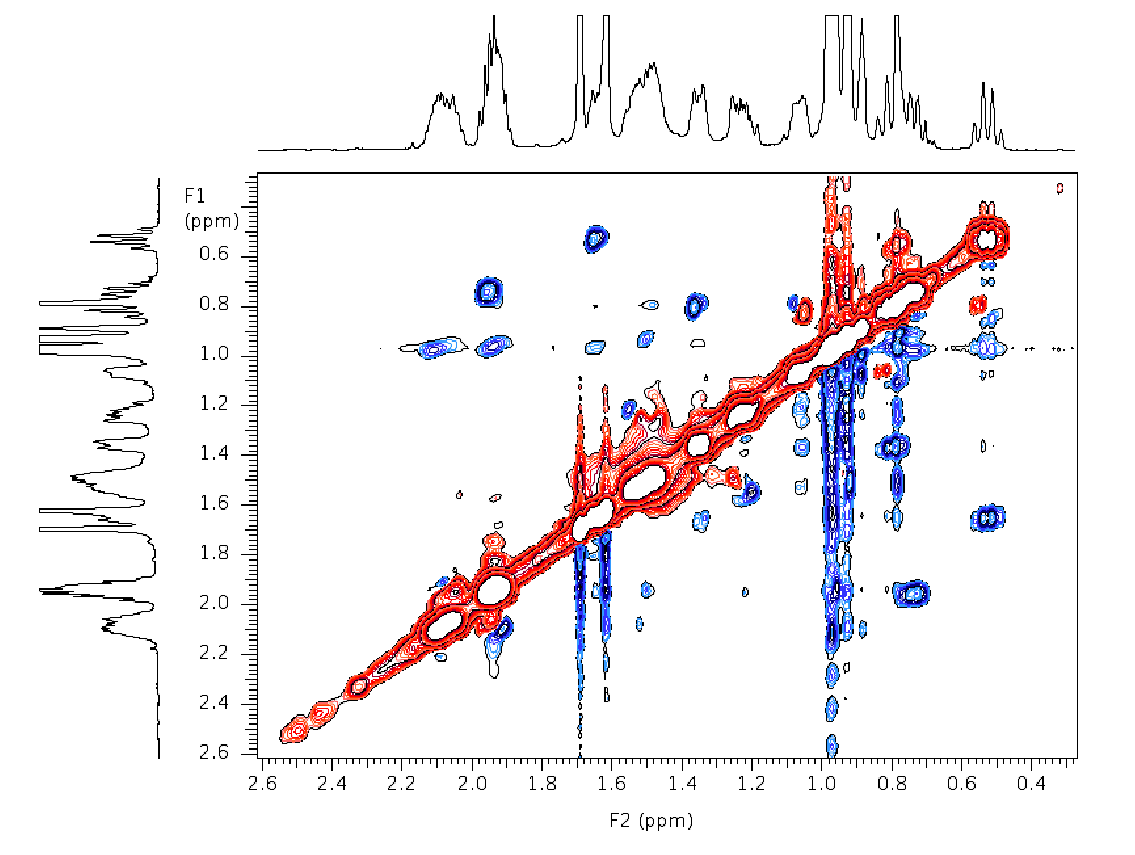


**Figure S9.** Partial NOESY (CDCl_3_) spectrum of compound **1**


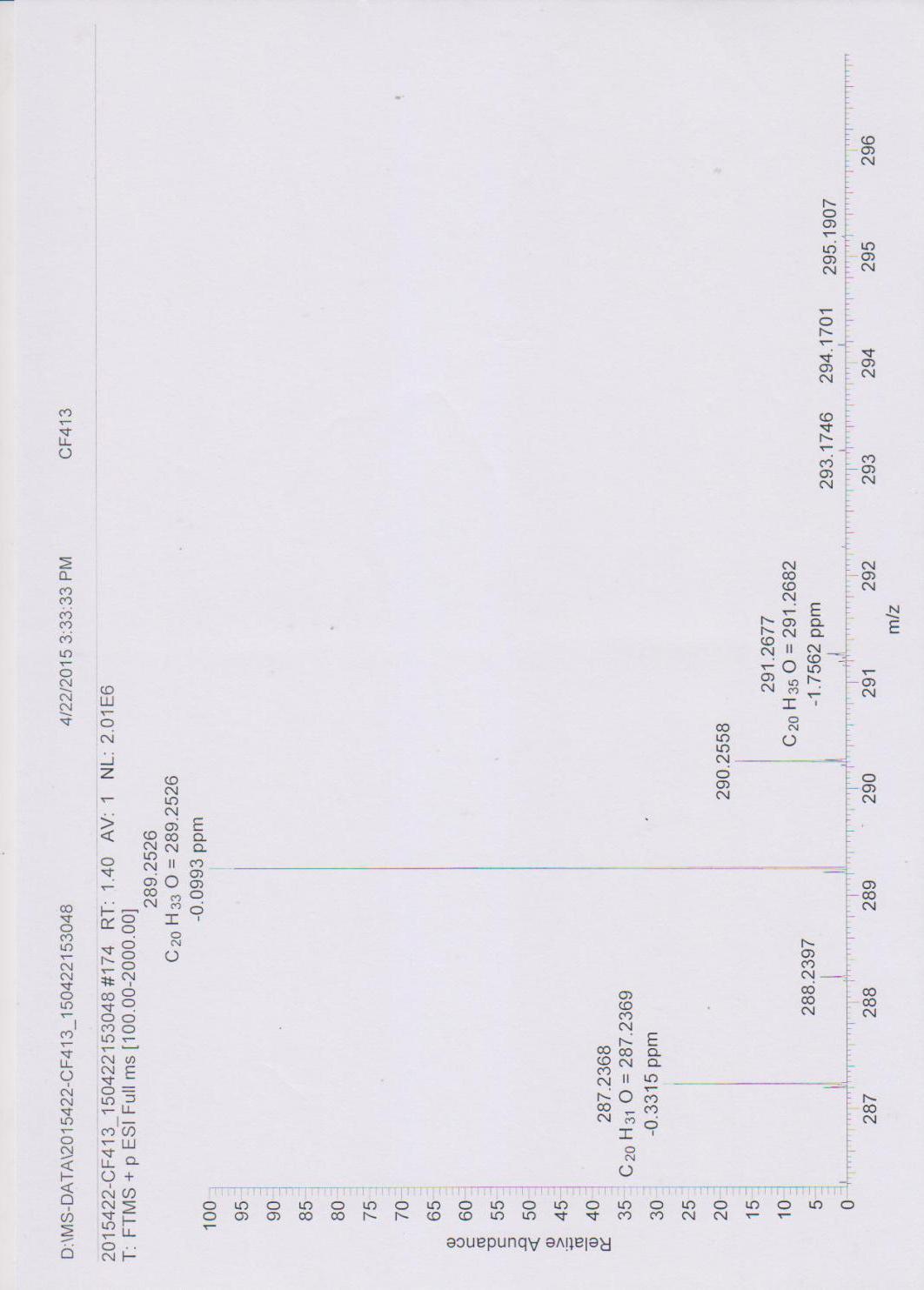


**Figure S10.** HRESIMS spectrum of compound **1**


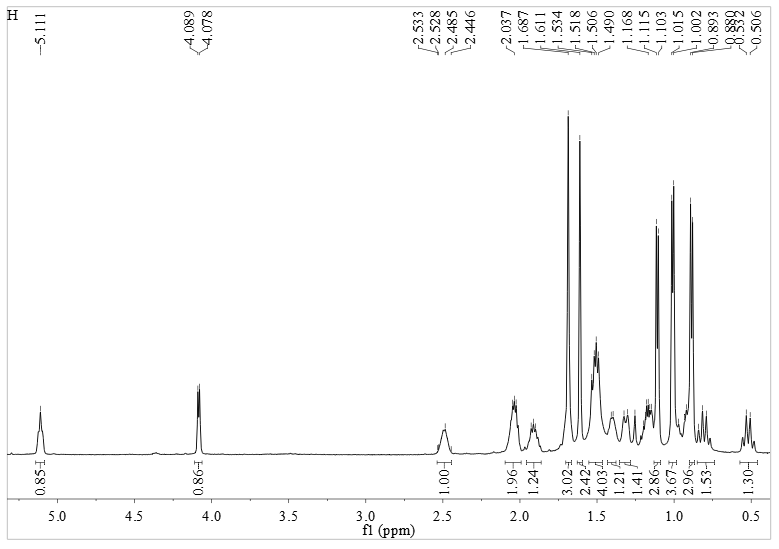


**Figure S11.** ^1^H NMR (500 MHz, CDCl_3_) spectrum of compound **2**


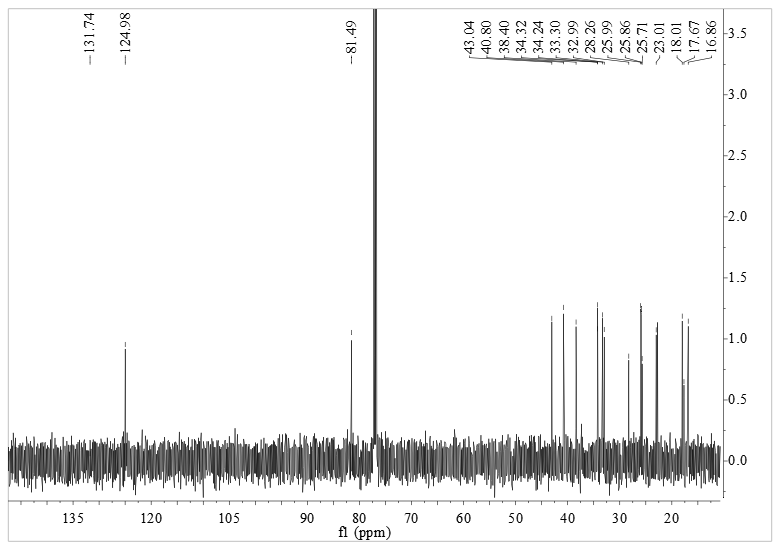


**Figure S12.** ^13^C NMR (125 MHz, CDCl_3_) spectrum of compound **2**


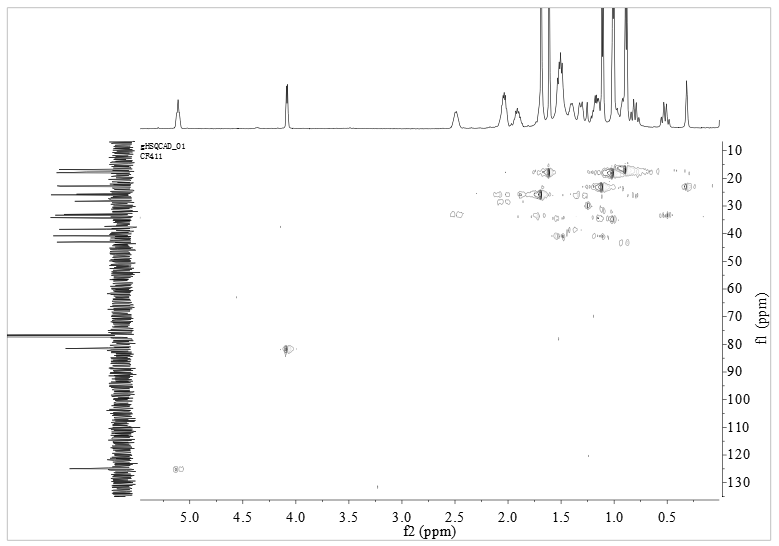


**Figure S13.** HMQC (CDCl_3_) spectrum of compound **2**


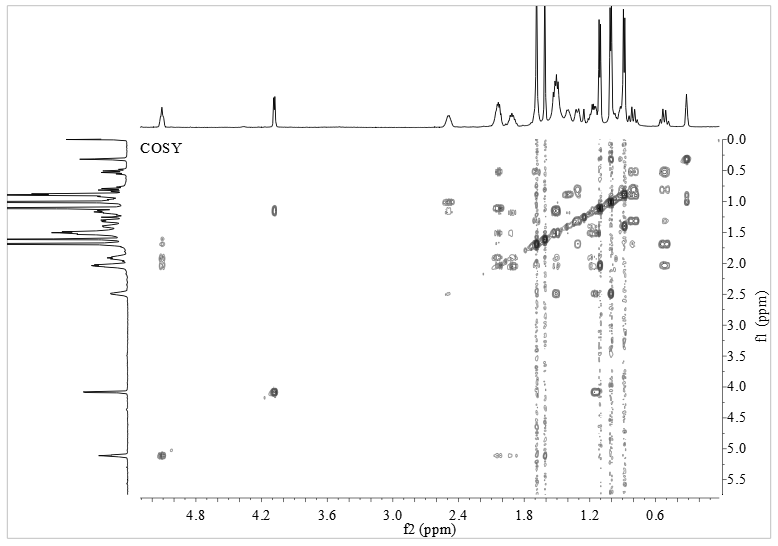


**Figure S14.** ^1^H-^1^H COSY (CDCl_3_) spectrum of compound **2**


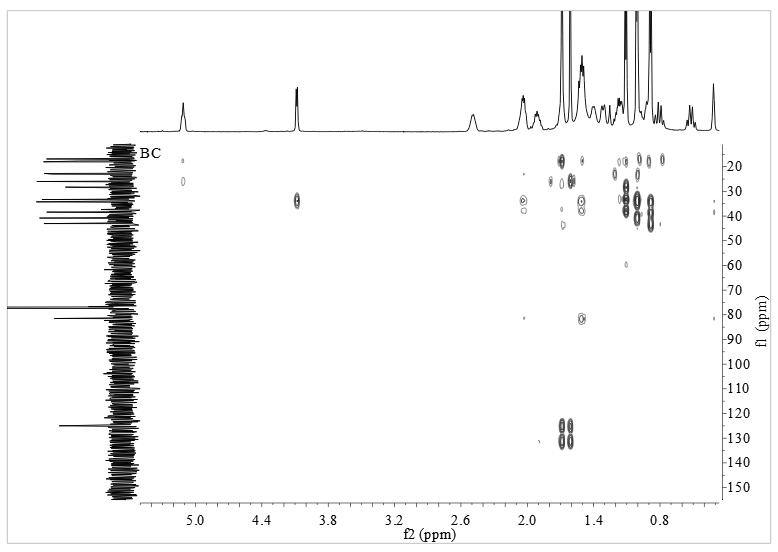


**Figure S15.** HMBC (CDCl_3_) spectrum of compound **2**


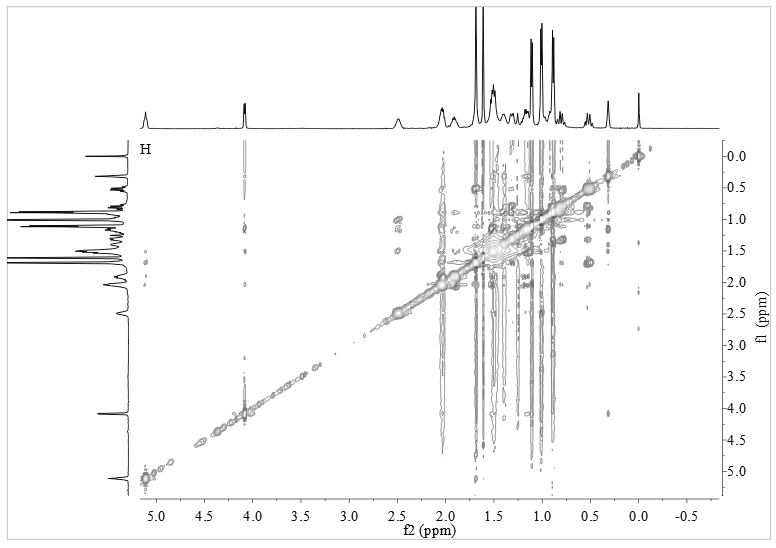


**Figure S16.** NOESY (CDCl_3_) spectrum of compound **2**

**Figure S17.** HRESIMS spectrum of compound **2**


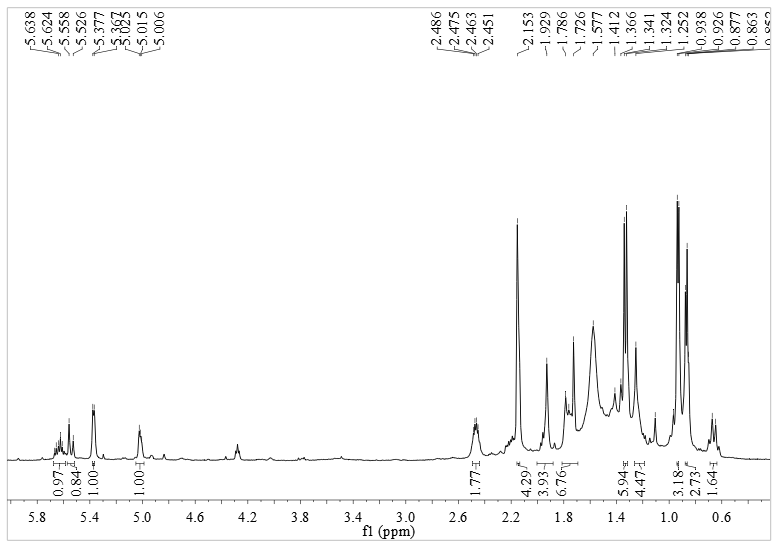


**Figure S18.** ^1^H NMR (500 MHz, CDCl_3_) spectrum of compound **3**


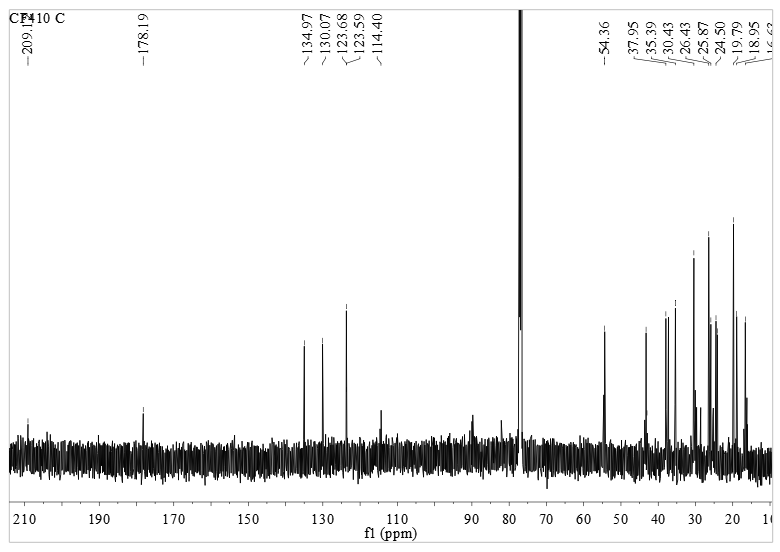


**Figure S19.** ^13^C NMR (125MHz, CDCl_3_) spectrum of compound **3**


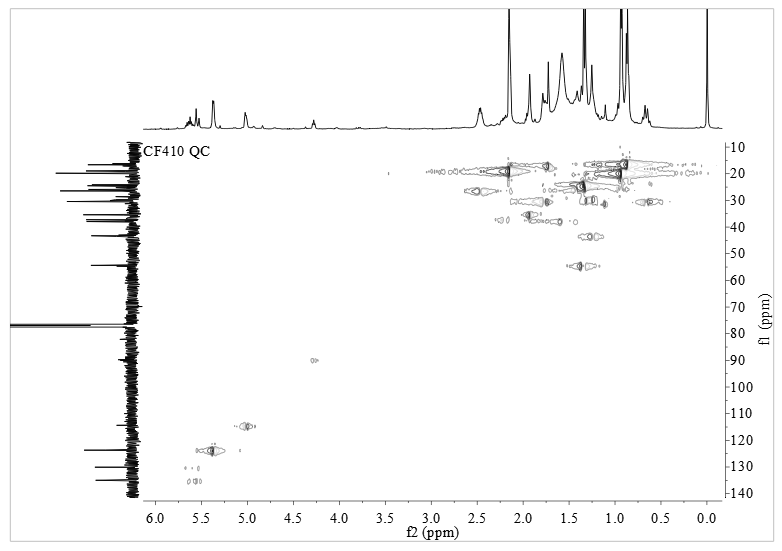


**Figure S20.** HMQC (CDCl_3_) spectrum of compound **3**


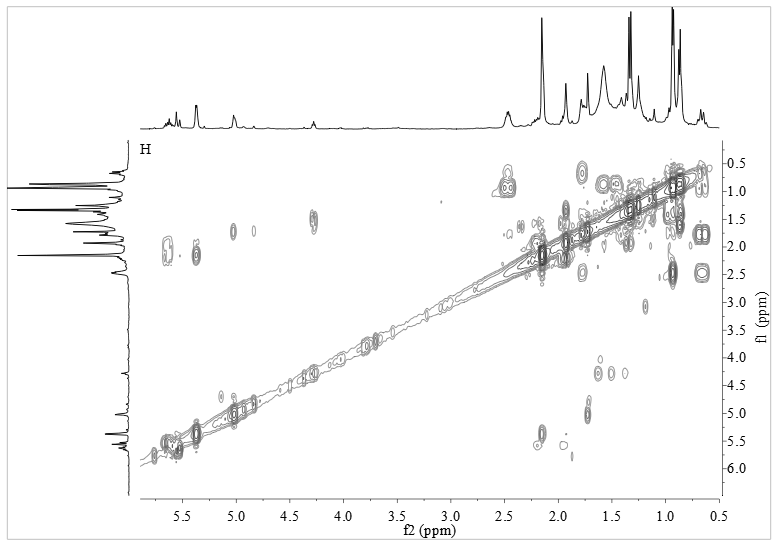


**Figure S21.** ^1^H-^1^H COSY (CDCl_3_) spectrum of compound **3**


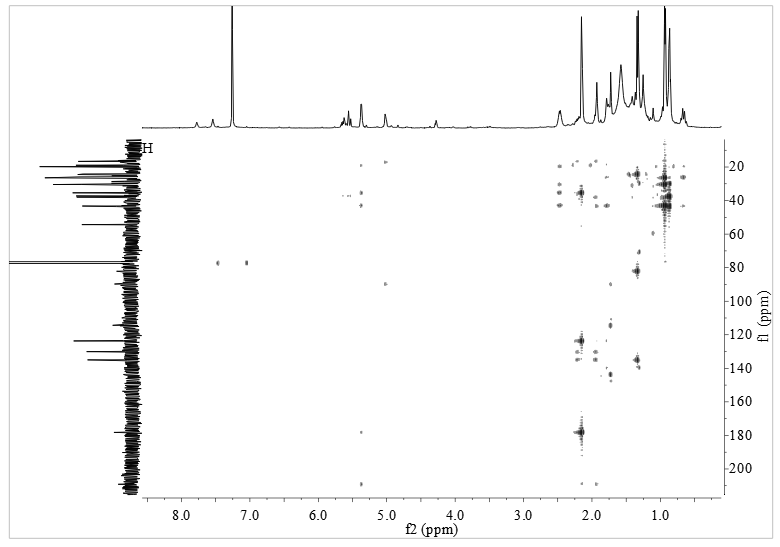


**Figure S22** HMBC (CDCl_3_) spectrum of compound **3**


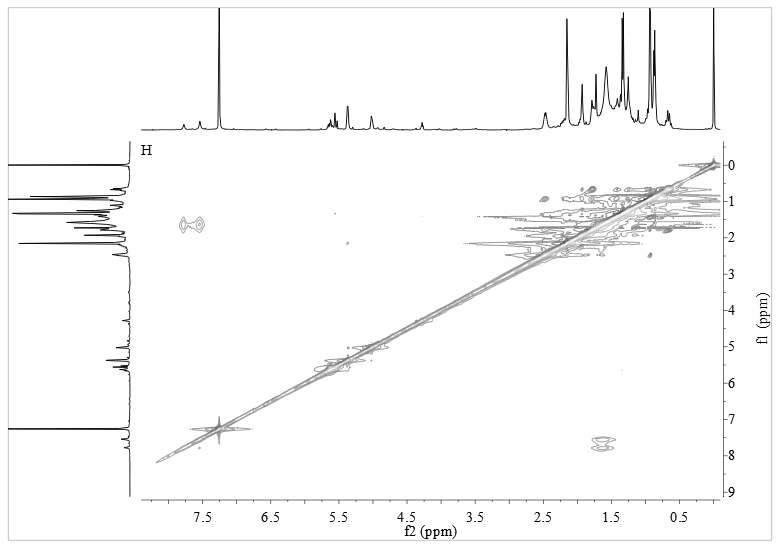


**Figure S23.** NOESY (CDCl_3_) spectrum of compound **3**

**Figure S24.** HRESIMS spectrum of compound **3**


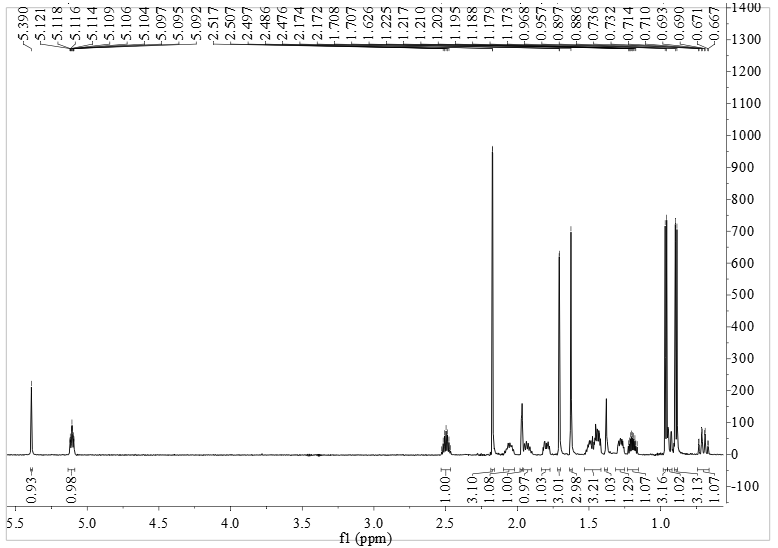


**Figure S25.** ^1^H NMR (500 MHz, CDCl_3_) spectrum of compound **4**


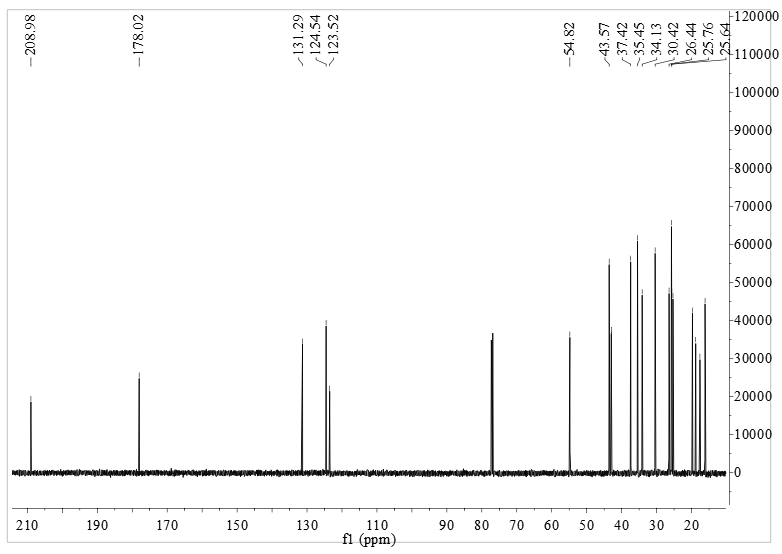


**Figure S26.** ^13^C NMR (125 MHz, CDCl_3_) spectrum of compound **4**


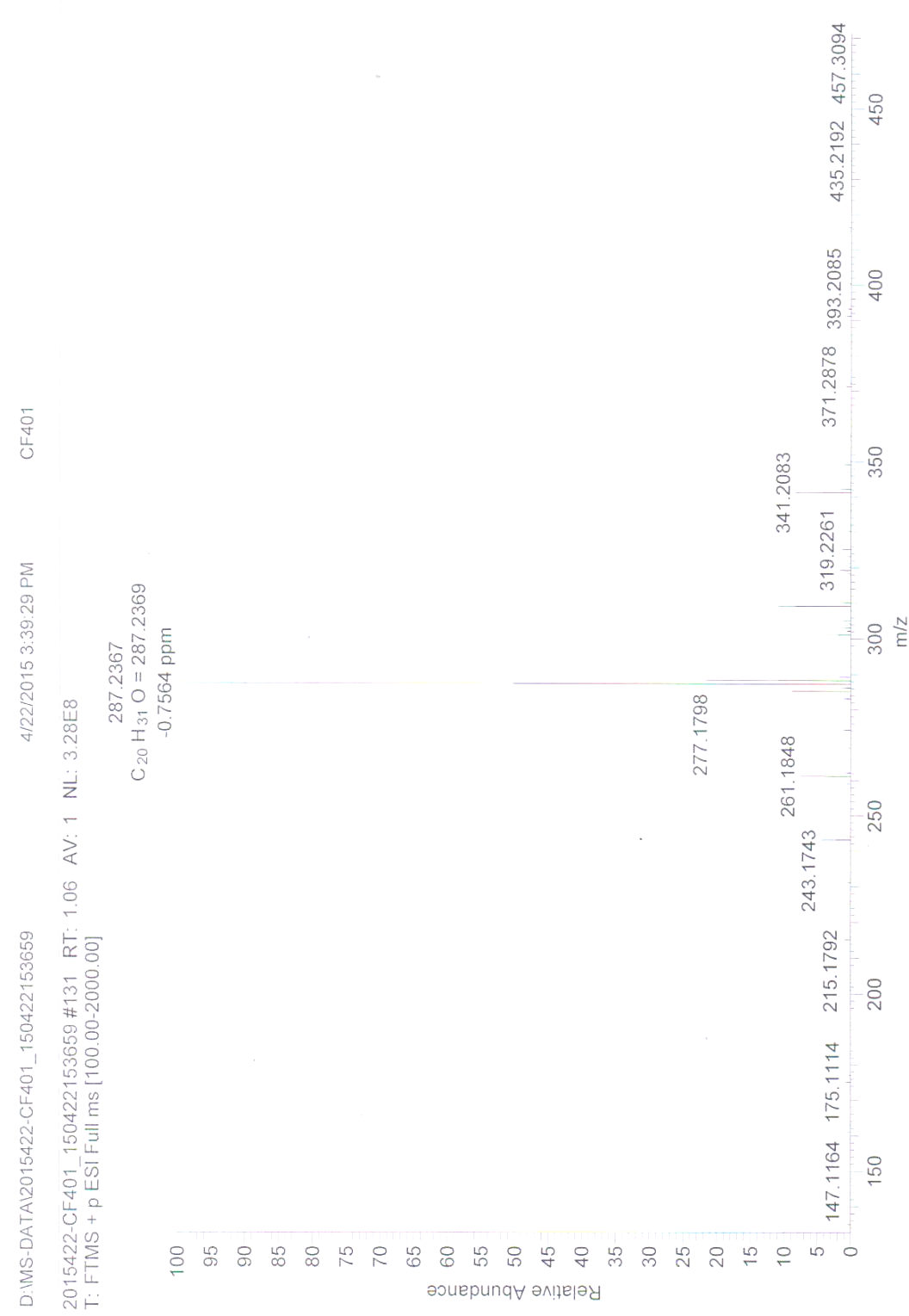


**Figure S27.** HRESIMS spectrum of compound **4**


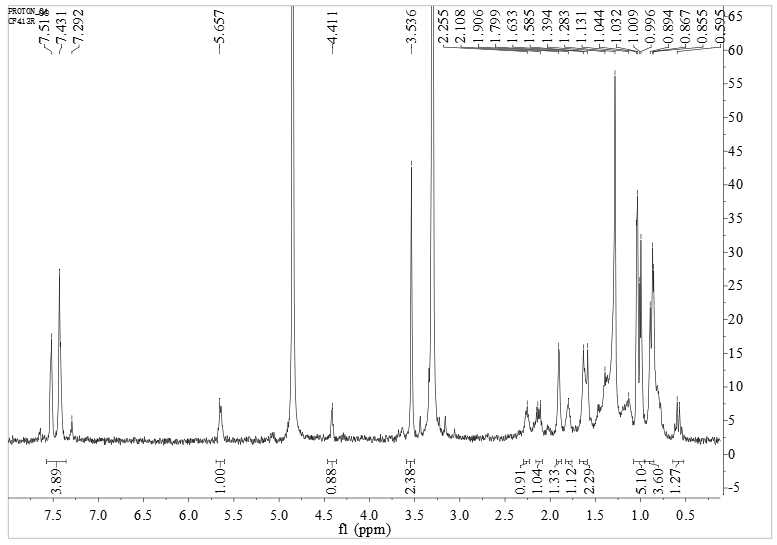


**Figure S28.** ^1^H NMR (500 MHz, CD_3_OD) spectrum of compound **1s**


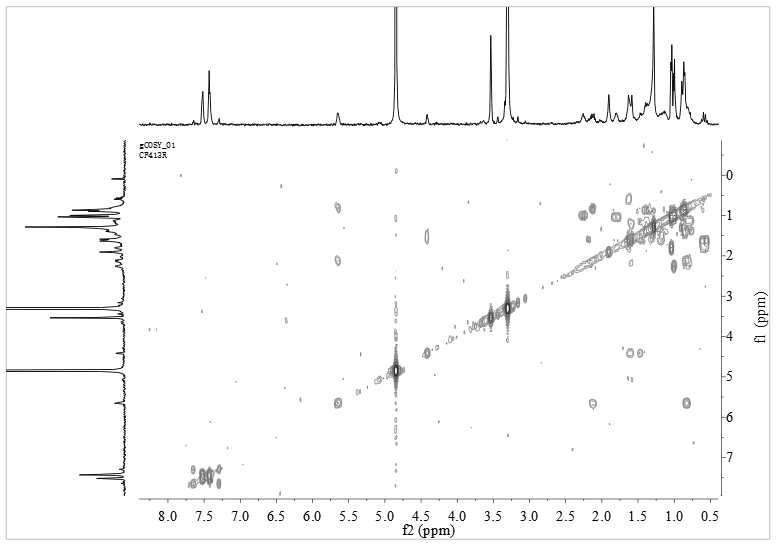


**Figure S29.** **^1^**H-^1^H COSY (CD_3_OD) spectrum of compound **1s**

**
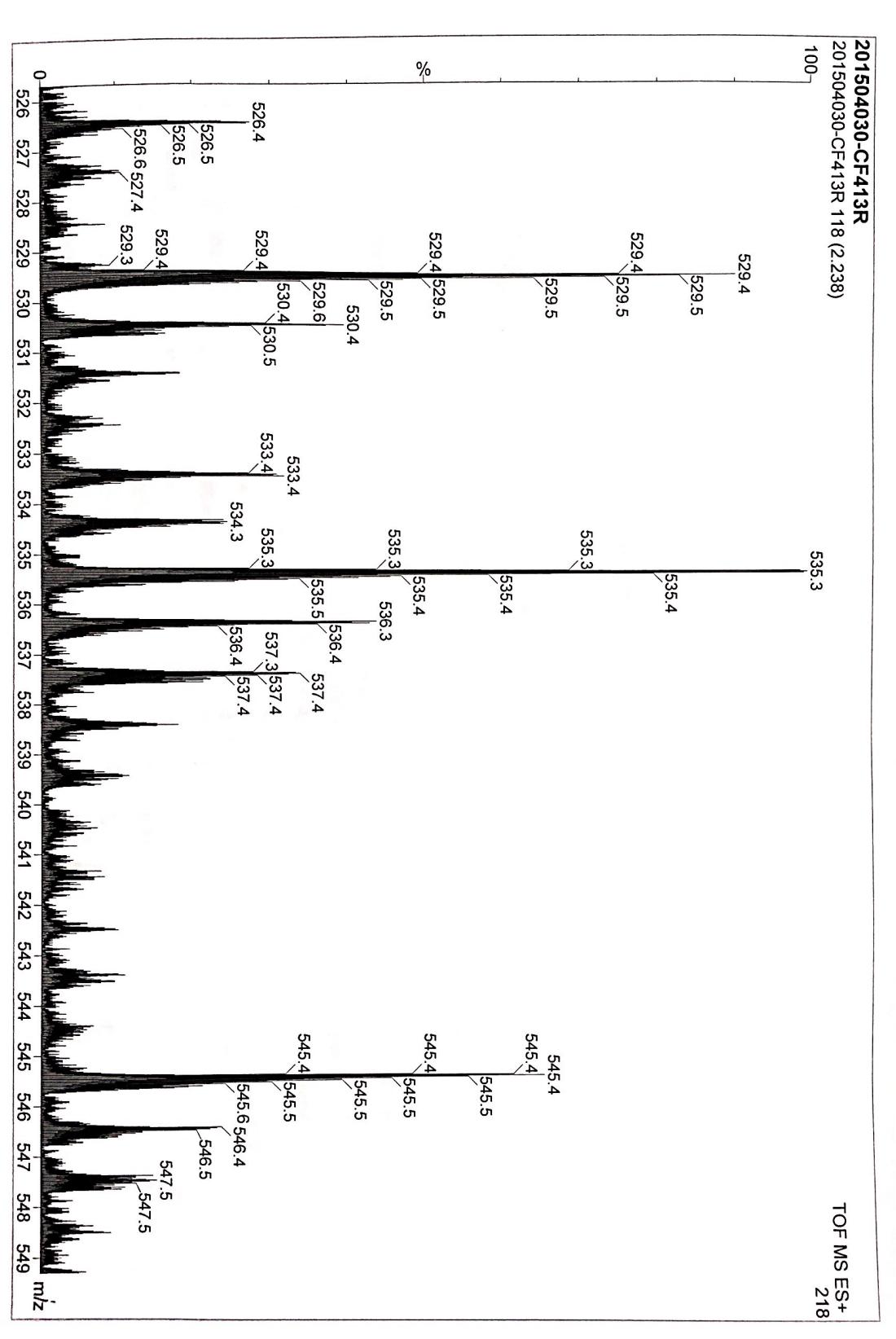
**

**Figure S30.** ESI-MS spectrum of compound **1s**


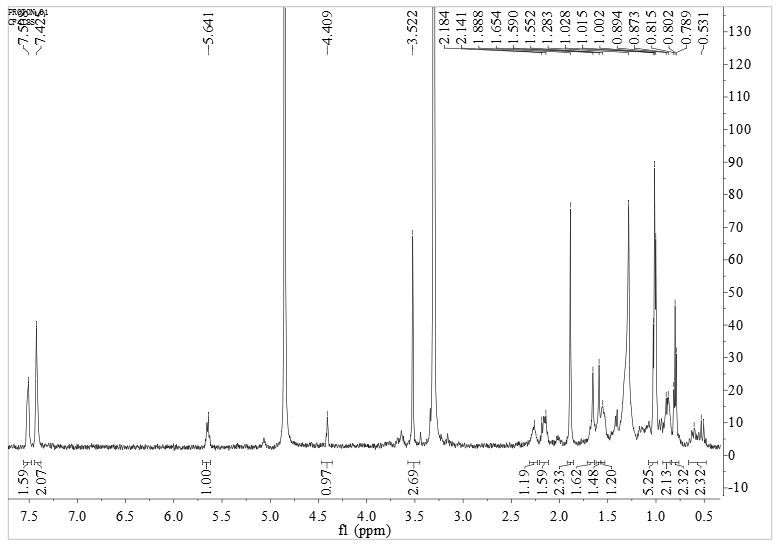


**Figure S31.** ^1^H NMR (500 MHz, CD_3_OD) spectrum of compound **1r**


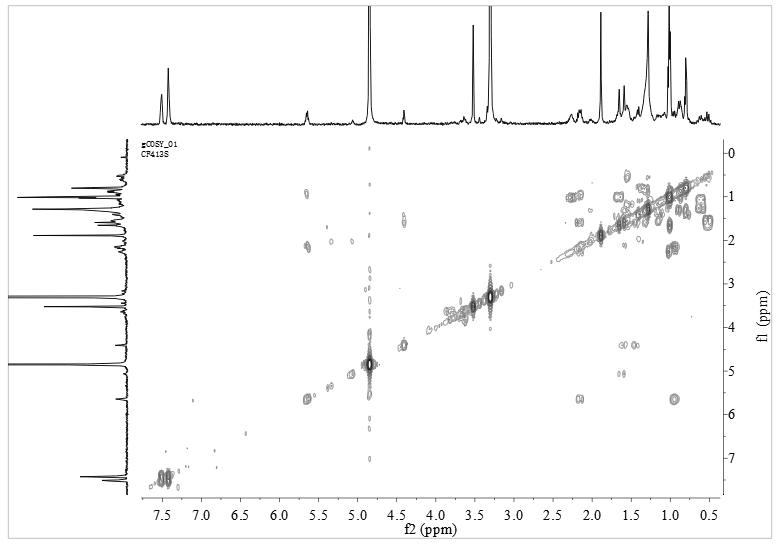


**Figure S32.** **^1^**H-^1^H COSY (CD_3_OD) spectrum of compound **1r**

**
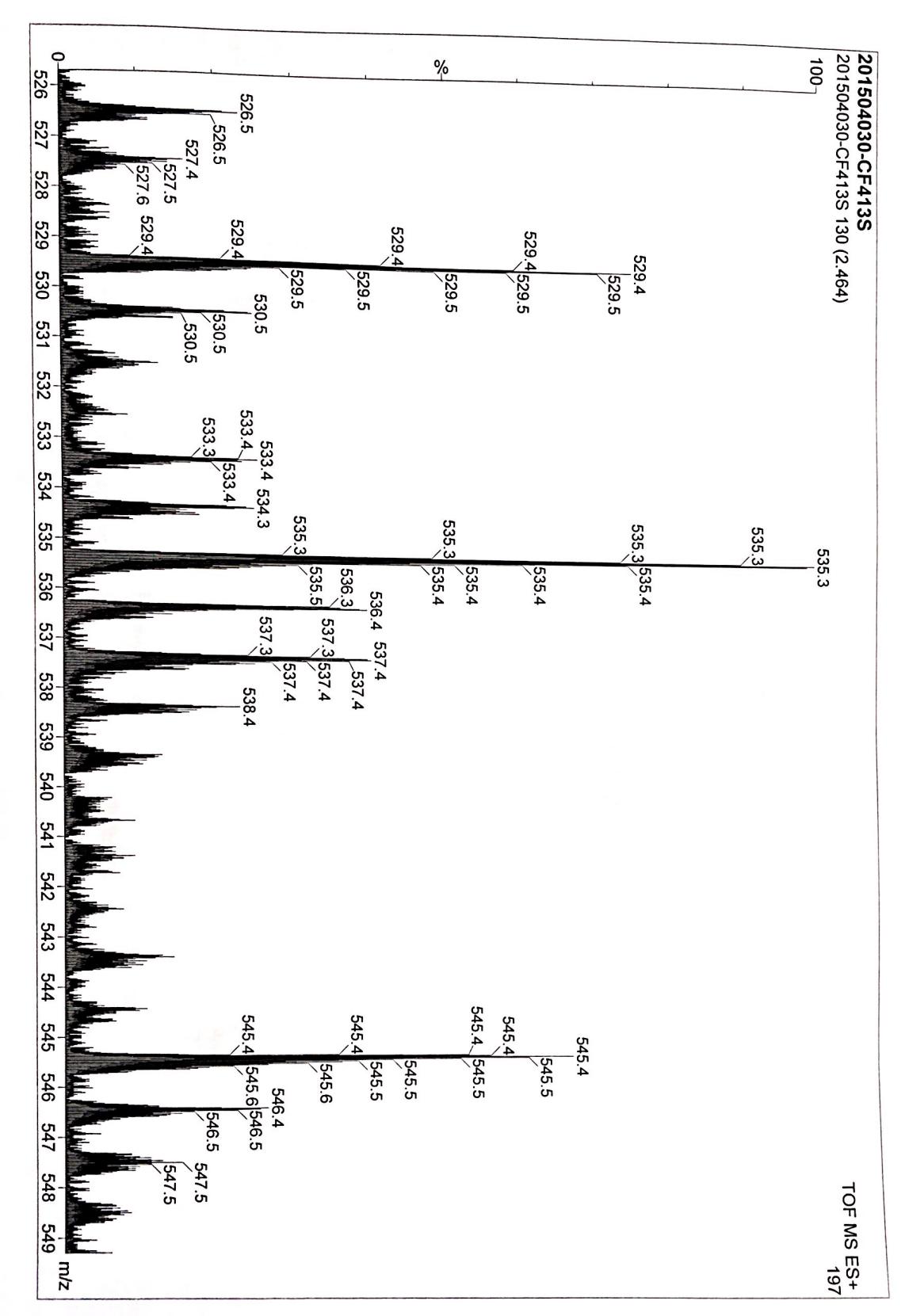
**

**Figure S33.** ESI-MS spectrum of compound **1r**

**Figure S34.** Ten lowest energy conformers for (1*S*,4*R*,5*R*,9*R*,10*S*,11*R*)-4

**
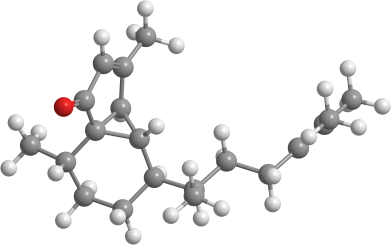

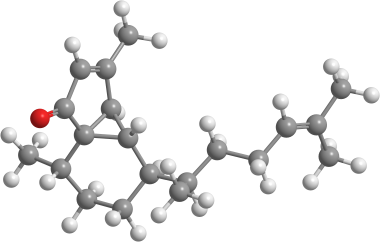

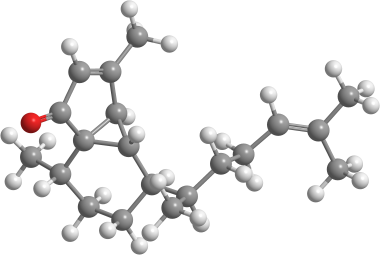
**

**C1 C2 C3**

**
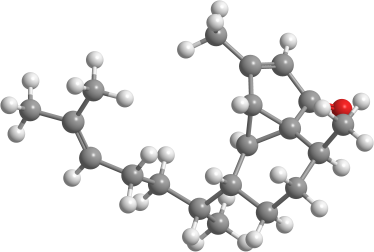

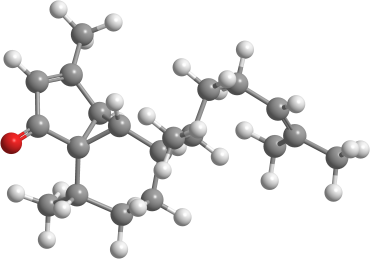

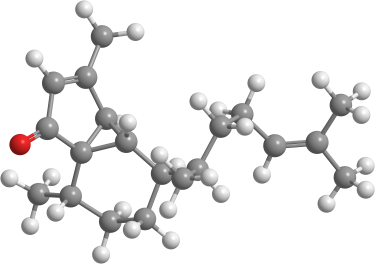
**

**C4 C5 C6**

**
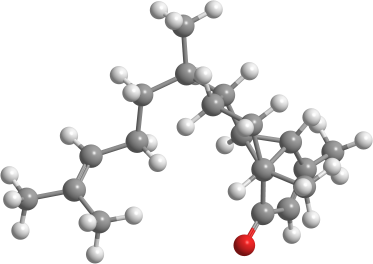

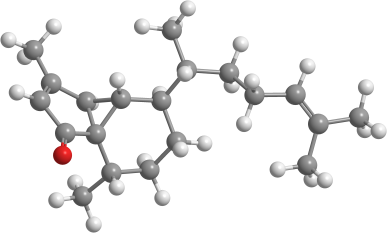

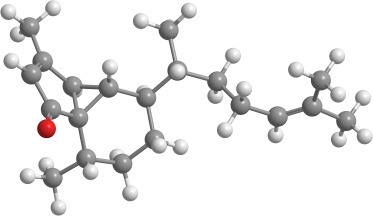
**

**C7 C8 C9**

**
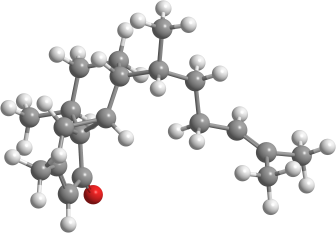
**

**C10**

**Figure S35.** Ninteen lowest energy conformers for (1*S*,4*R*,5*R*,9*R*,10*S*,11*S*)-**4**

**
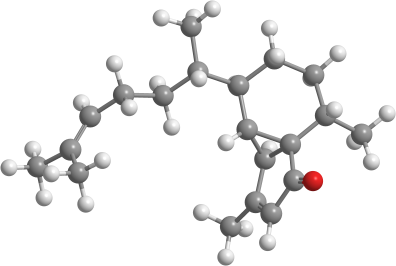

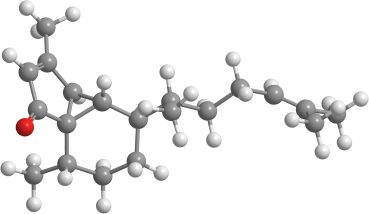

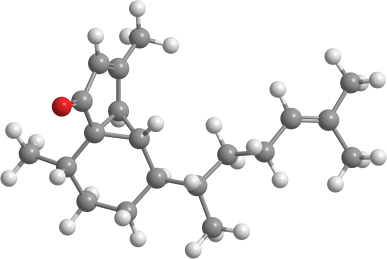
**

**C1 C2 C3**

**
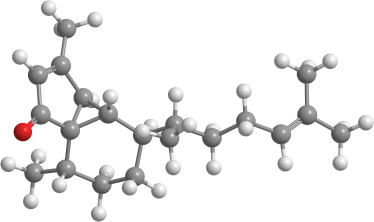

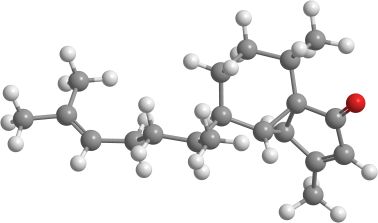

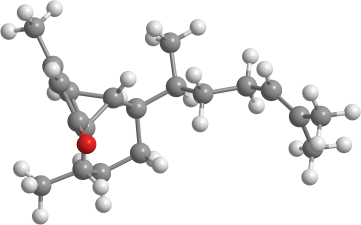
**

**C4 C5 C6**

**
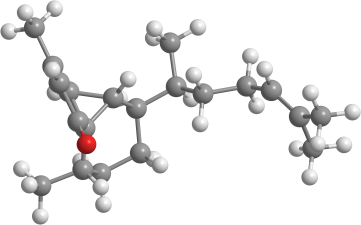

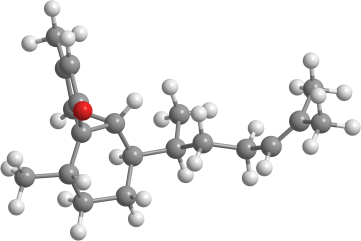

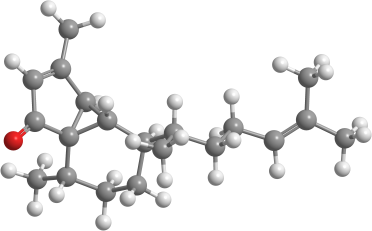
**

**C7 C8 C9**

**
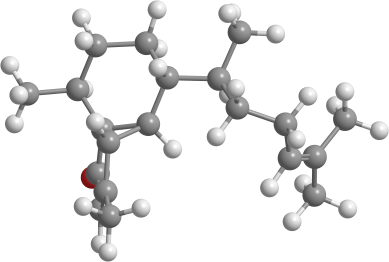

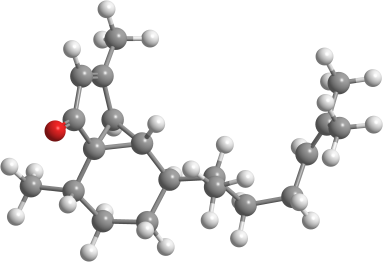

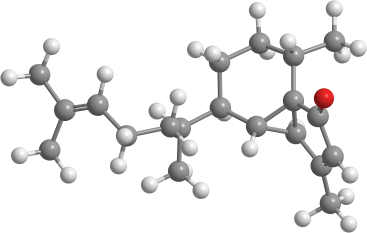
**

**C10 C11 C12**

**
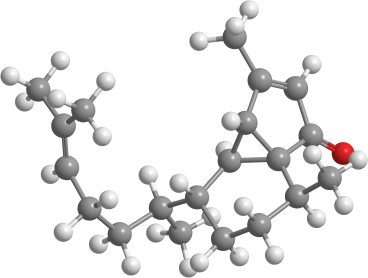

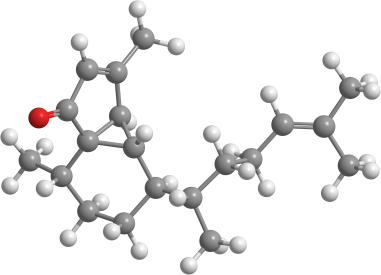

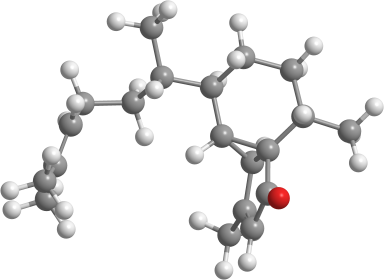
**

**C13 C14 C15**

**
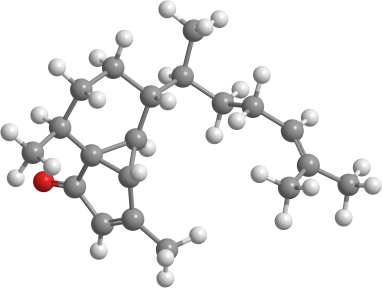

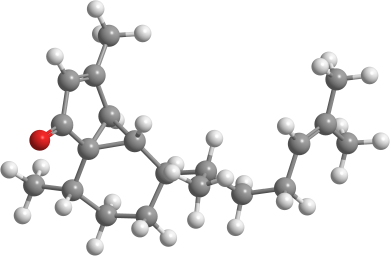

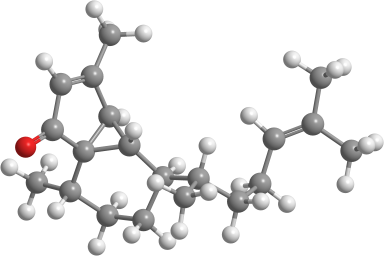
**

**C16 C17 C18**

**
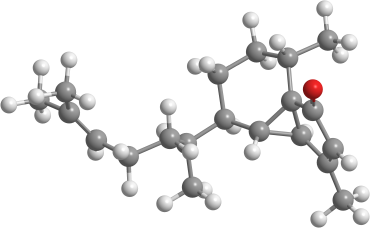
**

**C19**

**Table S1. Energy analysis for (1*S*,4*R*,5*R*,9*R*,10*S*,11*R*)-4**

| Conformers | HF | E | E*1060.475 | exp | % |
| --- | --- | --- | --- | --- | --- |
| C1 | -855.582941 | 0 | 0 | 1 | 0.238914 |
| C2 | -855.58278 | 0.000161 | 0.170843 | 0.842954 | 0.201394 |
| C3 | -855.582682 | 0.00026 | 0.275511 | 0.759184 | 0.18138 |
| C4 | -855.582498 | 0.000443 | 0.470109 | 0.624934 | 0.149306 |
| C5 | -855.581966 | 0.000976 | 1.034812 | 0.355293 | 0.084885 |
| C6 | -855.581248 | 0.001694 | 1.796233 | 0.165923 | 0.039641 |
| C7 | -855.580538 | 0.002403 | 2.548215 | 0.078221 | 0.018688 |
| C8 | -855.580492 | 0.002449 | 2.597315 | 0.074473 | 0.017793 |
| C9 | -855.58049 | 0.002451 | 2.599118 | 0.074339 | 0.017761 |
| C10 | -855.580255 | 0.002686 | 2.84833 | 0.057941 | 0.013843 |

**Table S2. Energy analysis for (1*S*,4*R*,5*R*,9*R*,10*S*,11*S*)-4**

| Conformers | HF | E | E*1060.475 | exp | % |
| --- | --- | --- | --- | --- | --- |
| C1 | -855.583019 | 0 | 0 | 1 | 0.105028 |
| C2 | -855.582997 | 0.000023 | 0.024073 | 0.976215 | 0.10253 |
| C3 | -855.582886 | 0.000133 | 0.140831 | 0.868636 | 0.091232 |
| C4 | -855.582825 | 0.000195 | 0.206262 | 0.81362 | 0.085453 |
| C5 | -855.582621 | 0.000398 | 0.422281 | 0.65555 | 0.068851 |
| C6 | -855.582534 | 0.000485 | 0.514649 | 0.597711 | 0.062777 |
| C7 | -855.582534 | 0.000485 | 0.514649 | 0.597711 | 0.062777 |
| C8 | -855.582525 | 0.000494 | 0.524193 | 0.592033 | 0.06218 |
| C9 | -855.582454 | 0.000566 | 0.599805 | 0.548919 | 0.057652 |
| C10 | -855.582355 | 0.000665 | 0.704792 | 0.494212 | 0.051906 |
| C11 | -855.582242 | 0.000777 | 0.824095 | 0.438632 | 0.046069 |
| C12 | -855.58199 | 0.00103 | 1.091865 | 0.33559 | 0.035247 |
| C13 | -855.581713 | 0.001307 | 1.385617 | 0.250169 | 0.026275 |
| C14 | -855.581622 | 0.001397 | 1.481908 | 0.227204 | 0.023863 |
| C15 | -855.581476 | 0.001543 | 1.636525 | 0.194655 | 0.020444 |
| C16 | -855.581466 | 0.001553 | 1.647342 | 0.192561 | 0.020224 |
| C17 | -855.581413 | 0.001606 | 1.703441 | 0.182056 | 0.019121 |
| C18 | -855.581098 | 0.001921 | 2.037491 | 0.130355 | 0.013691 |
| C19 | -855.58107 | 0.001949 | 2.066866 | 0.126582 | 0.013295 |

**Table S3. NMR spectroscopic data for compound 4^a^. ^a^Spectra measured at 500 MHz in CDCl_3_**

| **No.** | **^1^H** | **^13^C** |
| --- | --- | --- |
| 1 | 2.49, m | 26.4, CH |
| 2 | 1.80, m | 30.4, CH_2_ |
|  | 0.69, m |  |
| 3 | 1.44, m | 25.3, CH_2_ |
|  | 0.94, m |  |
| 4 | 1.28, m | 43.6, CH |
| 5 | 1.96, m | 35.5, CH |
| 6 | - | 178.0, C |
| 7 | 5.39, s | 123.5, CH |
| 8 | - | 209.0, C |
| 9 | - | 43.0, C |
| 10 | 1.38, t (2.8) | 54.8, CH |
| 11 | 1.49, m | 37.4, CH |
| 12 | 0.89, d (6.6) | 16.1, CH_3_ |
| 13 | 1.45, m | 34.2, CH_2_ |
|  | 1.20, m |  |
| 14 | 2.06, m | 25.8, CH_2_ |
|  | 1.94, m |  |
| 15 | 5.10, t (6.0) | 124.5, CH |
| 16 | - | 131.3, C |
| 17 | 1.63, s | 17.6, CH_3_ |
| 18 | 1.71, s | 25.6, CH_3_ |
| 19 | 0.96, d (6.5) | 19.8, CH_3_ |
| 20 | 2.17, s | 18.8, CH_3_ |
